# Sympathetic Overactivation Drives Neurogenic Alveolar Epithelial Pyroptosis via the PIEZO2‐ER Stress Pathway in Acute Lung Injury Following Intracerebral Hemorrhage

**DOI:** 10.1002/cns.71010

**Published:** 2026-07-06

**Authors:** Shuai Han, Zirui Wang, Qiuyue Zheng, Feng Guo, Yinggang Xiao, Zi Wang, Tianfeng Huang, Yongxin Liang, Bingchun Yan, Ju Gao

**Affiliations:** ^1^ Anesthesiology Department Northern Jiangsu People's Hospital Affiliated to Yangzhou University, Yangzhou University/the First School of Clinical Medicine, Faculty of Medicine, Yangzhou University Yangzhou Jiangsu China; ^2^ Peking University People's Hospital, Qingdao/Women and Children’s Hospital, Qingdao University Qingdao Shandong China

**Keywords:** acute lung injury, brain‐lung crosstalk, intracerebral hemorrhage, PIEZO2, stellate ganglion block, sympathetic overactivation

## Abstract

**Aims:**

Intracerebral hemorrhage (ICH) frequently triggers acute lung injury (ALI) via sympathetic overactivation. We aimed to investigate the neuroinflammatory mechanisms driving this brain‐lung crosstalk and evaluate targeted neuromodulatory interventions via the mechanosensor PIEZO2.

**Methods:**

We utilized an ICH mouse model and norepinephrine (NE)‐stimulated MLE12 cells. Pathological mechanisms were explored using transcriptomics, calcium imaging, and Vps35 knockdown. Therapeutic efficacies were assessed via central sympathetic blockade (stellate ganglion block, SGB) and peripheral PIEZO2 inhibition (D‐GsMTx4).

**Results:**

NE release exacerbated neurogenic ALI post‐ICH. Mechanistically, NE upregulated VPS35, which simultaneously promoted PIEZO2 membrane trafficking and impaired ATP2A2‐dependent endoplasmic reticulum (ER) calcium clearance. This induced massive intracellular calcium influx, triggering widespread ER stress and NLRP3/GSDMD‐mediated pyroptosis in alveolar epithelial cells. Vps35 knockdown attenuated these effects in vitro. Crucially, targeted interventions with SGB or D‐GsMTx4 successfully blocked this lethal central‐peripheral cascade, alleviating pulmonary pyroptosis and improving in vivo outcomes.

**Conclusion:**

Sympathetic overactivation drives neurogenic ALI post‐ICH via the VPS35/PIEZO2‐ER stress‐pyroptosis axis. Dual‐node neuromodulation (SGB or PIEZO2 inhibition) offers a promising therapeutic strategy for secondary multiorgan complications following acute brain injury.

## Graphical abstract

Following intracerebral hemorrhage, sympathetic overactivation drives acute lung injury via norepinephrine‐induced VPS35 upregulation. VPS35 promotes PIEZO2‐mediated calcium influx and impairs ATP2A2‐dependent ER calcium reuptake, triggering ER stress and NLRP3‐pyroptosis. Targeted interventions (stellate ganglion block or PIEZO2 inhibitor D‐GsMTx4) effectively halt this neurogenic cascade.

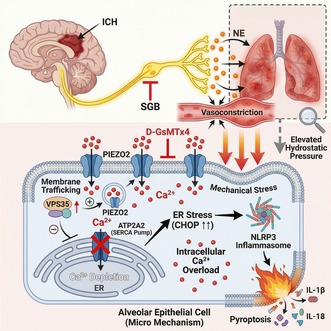

## 1 Introduction

Acute brain injury, including intracerebral hemorrhage (ICH), profoundly impacts peripheral organs, with the lungs being particularly vulnerable to this disrupted inter‐organ crosstalk [1, 2, 3]. Severe ICH frequently precipitates acute lung injury (ALI) or neurogenic pulmonary edema (NPE), leading to rapid clinical deterioration and mortality rates up to 60–100% [4, 5, 6, 7, 8]. Although delayed systemic inflammation contributes to ICH‐induced lung injury [1, 9], its rapid onset strongly implicates immediate neurogenic triggers, specifically massive catecholamine release resulting from profound sympathetic overactivation [10]. However, the precise intracellular networks mediating this early brain‐lung crosstalk remain incompletely understood.

Following acute brain injury, sympathetic hyperexcitability induces intense hemodynamic fluctuations, imposing extreme mechanical stress on the alveolar epithelium [11, 12]. To adapt to this physical tension, alveolar cells rely on mechanosensitive ion channels. Notably, PIEZO2 functions as a central mechanotransducer, converting physical forces into intracellular signals via rapid calcium influx [13, 14, 15]. Pathologically, mechanically‐driven calcium overload disrupts intracellular homeostasis, precipitating endoplasmic reticulum (ER) stress. This cascade subsequently activates the NLRP3/Caspase‐1/GSDMD pathway, culminating in inflammatory pyroptosis [16]. Yet, exactly how PIEZO2‐mediated mechanotransduction bridges sympathetic neuro‐signals and alveolar pyroptosis during neurogenic lung injury remains unclear.

Sympathetic hyperactivity is the primary initiator of this aberrant brain‐lung communication post‐ICH [17, 18]. To investigate the interplay between central sympathetic overactivation, mechanosensor dynamics, and alveolar calcium homeostasis, we established an in vivo ICH mouse model alongside an in vitro norepinephrine (NE)‐stimulated MLE‐12 cell model. Furthermore, we assessed the therapeutic potential of dual‐node neuromodulation: Targeting central sympathetic hyperexcitability via stellate ganglion block (SGB) and inhibiting peripheral mechanosensor overactivation with a specific PIEZO2 blocker (D‐GsMTx4). Ultimately, our findings aim to elucidate a novel neuro‐pulmonary inflammatory axis and validate targeted therapeutics for post‐ICH systemic complications.

## 2 Methods

### 2.1 Study Design

This study investigated the mechanisms of intracerebral hemorrhage (ICH)‐induced acute lung injury (ALI) and evaluated neuromodulatory interventions targeting sympathetic overactivation and PIEZO2. Adult male C57BL/6J mice were used to minimize sex‐hormone confounding. In vivo experiments included (i) time‐course characterization after ICH, (ii) SGB intervention, and (iii) PIEZO2 inhibition with D‐GsMTx4. In vitro mechanistic studies used norepinephrine (NE)‐stimulated MLE‐12 cells with Vps35 knockdown. Randomization and blind outcome assessments were applied where feasible. Detailed procedures are provided in Supplementary Methods.

### 2.2 Experimental Animals

The study was conducted in accordance with the National Institutes of Health Guide for the Care and Use of Laboratory Animals. Adult male C57BL/6J mice, weighing 20–25 g, were obtained from the Animal Experimental Center of Yangzhou University. Animals were housed under a standard 12‐h light/dark cycle and provided ad libitum access to food and water. Mice were assigned to experimental groups using a random number generator. The investigators performing outcome assessments were blind to these group allocations. Detailed animal husbandry and perioperative care are provided in Supplementary Methods.

### 2.3 ICH Model

To induce the murine ICH model [19], mice were subjected to a stereotaxic infusion of bacterial collagenase type IV. Mice were first anesthetized via 2% isoflurane inhalation and subsequently secured in a stereotaxic apparatus (Model 68,801, RWD Life Science, Shenzhen, China). A micro‐burr hole was drilled over the right hemisphere after exposing the skull. A Hamilton syringe was then advanced into the right basal ganglia. The stereotaxic coordinates were 0.2 mm posterior and 2.2 mm lateral to the bregma, at a depth of 3.5 mm from the dura. A total volume of 0.3 μL saline containing 0.03 U of collagenase was microinjected at a constant rate of 0.06 μL/min. To minimize potential solution reflux along the needle tract, the syringe was left in situ for 10 min before gradual withdrawal. Mice in the sham group underwent identical surgical procedures but received an equal volume of phosphate‐buffered saline (PBS). To assess the temporal profile of pulmonary damage, lung tissues were collected and evaluated at various intervals (6 h, 12 h, 1 day, 3 days, and 7 days) post‐hemorrhage.

### 2.4 Stellate Ganglion Block (SGB)

To investigate the role of the sympathetic nervous system in ICH‐induced lung injury, mice were randomly allocated into four experimental groups: Sham+NC, ICH + NC, Sham+SGB, and ICH + SGB. The SGB procedure was performed at 1 h, 24 h, and 48 h post‐ICH induction. Mice were anesthetized with 2% isoflurane and positioned prone on a surgical platform. The cartilaginous tip of the seventh cervical vertebra (C7) spinous process was identified by palpation. A microsyringe needle was advanced anteriorly along the right parasagittal plane of C7. When a loss of resistance was felt, suggesting the needle tip had bypassed the vertebral body, the needle was slightly withdrawn by approximately 0.5 mm. After aspiration to confirm the absence of blood or cerebrospinal fluid, 40 μL of 0.25% ropivacaine was slowly injected into the target region to induce sympathetic blockade. Mice in the NC groups received an equivalent volume of normal saline. Successful SGB was confirmed upon recovery from anesthesia by the presence of unilateral Horner's syndrome on the ipsilateral side. This syndrome, characterized by ptosis, enophthalmos, and miosis [20], was evaluated as a qualitative “yes/no” assessment by visually comparing the ipsilateral eye with the contralateral unaffected eye. Mice failing to exhibit these obvious clinical signs were considered to have unsuccessful blocks and were excluded from subsequent analyses.

### 2.5 PIEZO2 Inhibition (D‐GsMTx4)

To inhibit PIEZO2 in vivo, D‐GsMTx4, a widely used mechanosensitive ion channel inhibitor, was administered intraperitoneally (i.p.) at a dose of 0.5 mg/kg (or vehicle) at 1, 24, and 48 h post‐ICH. This specific dosage and administration route were selected based on recent in vivo studies demonstrating its efficacy and safety in systemically modulating PIEZO‐dependent signaling [21], as well as its established application in targeting PIEZO2‐related neural injury models [22]. Detailed information regarding the drug formulation and administration is provided in the Supplementary Methods.

### 2.6 Neurobehavioral Assessment

Neurological function was evaluated using the Morris Water Maze (MWM) at day 14 post‐ICH. Detailed procedures are provided in Supplementary Methods.

### 2.7 ECG And Heart Rate Variability

Autonomic function and sympathetic tone were assessed using electrocardiography and heart rate variability (HRV) analysis [23]. Detailed procedures are provided in Supplementary Methods.

### 2.8 Pulmonary Function Testing and Chest X‐Ray Imaging

Pulmonary mechanics were measured using plethysmography at the indicated time point(s) after ICH. Chest radiography was performed to evaluate lung involvement after ICH. Detailed procedures are provided in Supplementary Methods.

### 2.9 Brain Water Content and Lung Wet/Dry Ratio

Brain water content and lung wet/dry (W/D) ratios were measured to evaluate tissue edema. Detailed procedures are provided in Supplementary Methods.

### 2.10 Histology

Brain and lung tissues were fixed in 4% paraformaldehyde, embedded in paraffin, and cut into 5‐μm sections. Brain sections were stained with Hematoxylin and Eosin (HE) and Nissl, while lung sections were stained with HE alone. Lung injury was evaluated in a blind manner using a standardized scoring system. This system incorporated assessments of alveolar congestion, hemorrhage, neutrophil infiltration, and alveolar wall thickening.

### 2.11 RNA Sequencing and Bioinformatics Analysis

RNA sequencing of lung tissue was performed on an Illumina platform. Differentially expressed genes were analyzed, followed by GO and KEGG enrichment analyses to identify key pathways.

### 2.12 Cell Culture and NE Stimulation

The murine lung epithelial‐12 (MLE‐12) cell line, a widely used model of mouse alveolar type II epithelial cells, was utilized in this study to investigate alveolar injury in vitro. MLE‐12 cells were cultured under standard conditions and stimulated with NE (1 μM or 10 μM) to model sympathetic overactivation [24]. Detailed culture conditions and treatment schedules are provided in the Supplementary Methods.

### 2.13 siRNA Transfection

For Vps35 silencing, cells were transfected with one of three Vps35‐specific small interfering RNAs (si‐Vps35 1–3) or a GFP‐conjugated scrambled negative control siRNA (Scr‐siRNA), all synthesized by GeneChem (Shanghai, China). Transfections were performed using Lipofectamine 3000 according to the manufacturer's instructions. The specific oligonucleotide sequences are listed in Table S1. Transfection efficiency was assessed 24 h post‐transfection using GFP fluorescence microscopy. Knockdown efficiency was subsequently validated via quantitative RT‐PCR and Western blotting.

### 2.14 Intracellular Calcium Measurements

Intracellular Ca^2+^ levels were quantified by flow cytometry using Fluo‐3 AM, and total cellular calcium was measured using colorimetric assays. Detailed procedures are provided in Supplementary Methods.

### 2.15 Quantification of Cytokines and Catecholamines

Inflammatory cytokines and catecholamines were quantified using ELISA‐based assays. Absorbance readings and sample preparations were conducted according to the manufacturer's protocols.

### 2.16 Cell Viability and Pyroptosis Assessment

Cell viability and membrane integrity were evaluated using CCK‐8 and LDH release assays, and pyroptosis‐related markers were assessed accordingly. Detailed procedures are provided in Supplementary Methods.

### 2.17 Immunofluorescence Staining

Immunofluorescence was performed to assess protein localization and expression in tissues and cells. Detailed antibody information and procedures are provided in Supplementary Methods.

### 2.18 RT‐qPCR Analysis

Gene expression was quantified using RT‐qPCR. All specific primer sequences are detailed in Table S2. Cycling conditions are provided in Supplementary Methods.

### 2.19 Western Blotting

Protein expression was analyzed by Western blotting for mechanotransduction, ER stress, and pyroptosis‐related targets. Detailed antibody information and procedures are provided in Supplementary Methods.

### 2.20 Statistical Analysis

Data are presented as mean ± SD. Two‐group comparisons used unpaired Student's *t*‐tests. Multiple‐group comparisons used one‐way ANOVA with Tukey's *post hoc* test, and time‐course data used two‐way repeated‐measures ANOVA with Bonferroni's *post hoc* test. *p* < 0.05 was considered statistically significant.

## 3 Results

### 3.1 ICH Induces Cognitive Deficits and Progressive Acute Lung Injury Peaking at Day 3

**FIGURE 1:**
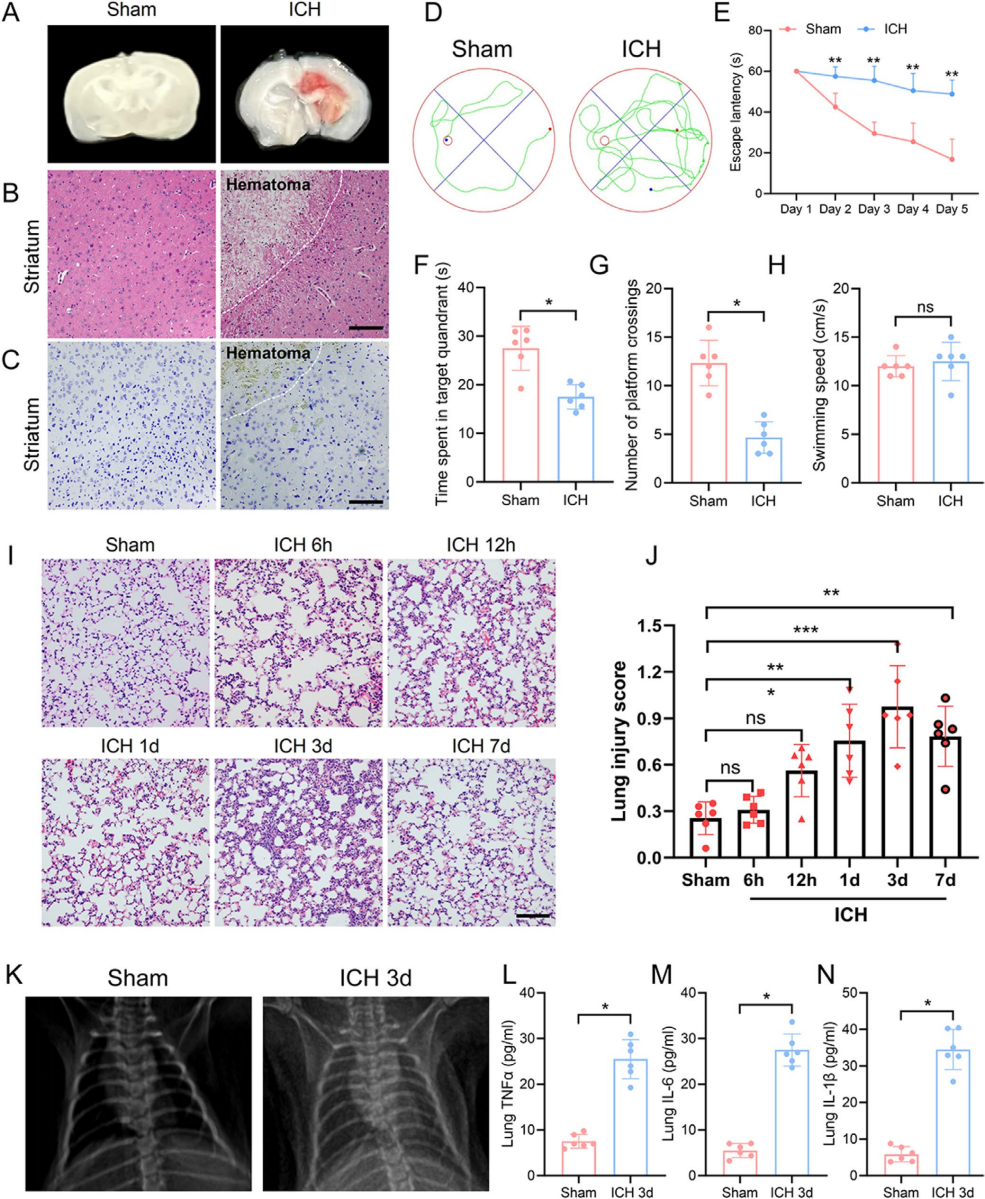
Intracerebral hemorrhage (ICH) is associated with neurocognitive deficits and progressive acute lung injury peaking at day 3. (A) Representative coronal brain sections indicating successful hematoma formation (red) post‐ICH. (B, C) Representative images of HE (B) and Nissl (C) staining in the striatum (peri‐hematomal region for the ICH group) of mice from the Sham and ICH groups. Scale bars, 100 μm. (D–H) Evaluation of spatial learning and memory via the Morris Water Maze (MWM) test, showing representative trajectories (D), escape latency (E), time in the target quadrant (F), platform crossings (G), and swimming speed (H). (I) Representative HE images of lung tissues at the indicated time points post‐ICH, identifying day 3 as the peak of pathological injury. Scale bars, 100 μm. (J) Quantification of lung injury scores based on HE staining. (K) Representative chest X‐ray images revealing diffuse pulmonary inflammatory infiltrates at day 3 post‐ICH. (L–N) ELISA quantification of pro‐inflammatory cytokines TNF‐α (L), IL‐6 (M), and IL‐1β (N) in lung homogenates. Data are presented as mean ± SD. *n* = 3 (A–C, K) or *n* = 6 (D–J, L–N) biologically independent animals per group. Statistical significance was determined by unpaired two‐tailed Student's *t*‐test (F–H, L–N), one‐way ANOVA with Tukey's *post hoc* test (J), or two‐way repeated‐measures ANOVA with Bonferroni's *post hoc* test (E). *p < 0.05, **p < 0.01, ***p < 0.001 vs. Sham; ns, not significant.

To investigate brain‐lung crosstalk, we established a murine model of ICH. Model induction was macroscopically confirmed by distinct hematomas in coronal brain sections (Figure 1A). Compared to the Sham group, histological evaluations via HE and Nissl staining of ICH mice revealed localized pathological damage, neuronal loss, and structural disorganization in the peri‐hematomal region (Figure 1B,C). This primary brain injury was associated with spatial learning and memory deficits in the MWM test. Specifically, ICH mice exhibited chaotic swimming trajectories, prolonged escape latency, reduced time in the target quadrant, and fewer platform crossings. Average swimming speed remained unaltered, suggesting that these deficits were not primarily due to motor dysfunction (Figure 1D–H).

Next, we characterized the temporal progression of secondary pulmonary alterations at 6 h, 12 h, 1 day, 3 days, and 7 days post‐ICH. Histological examinations and lung injury scores indicated progressive pulmonary edema, alveolar septal thickening, and inflammatory infiltration. These pathological changes peaked at day 3 post‐ICH (Figure 1I,J). Consistently, quantitative assessments of pulmonary edema, including BALF protein concentration and lung W/D weight ratio, also reached their maximum on day 3 (Figure S1A,B). Thus, the 3‐day post‐ICH time point was selected for subsequent mechanistic investigations.

At day 3 post‐ICH, chest X‐ray imaging revealed diffuse inflammatory infiltrates across the lung fields of ICH mice (Figure 1K). This local inflammatory response was accompanied by elevated levels of pro‐inflammatory cytokines (TNF‐α, IL‐6, and IL‐1β) in lung tissues (Figure 1L–N) and serum (Figure S1C–E). Additionally, Evans blue dye extravasation assays indicated increased alveolar‐capillary barrier permeability (Figure S1F). This was associated with reduced expression of tight junction proteins, ZO‐1 and Claudin‐5, in the pulmonary epithelium (Figure S1G). Together, these structural and inflammatory alterations might contribute to the observed decline in overall pulmonary function in ICH mice (Figure S1H).

### 3.2 Transcriptomic Profiling Reveals Sympathetic Overactivation and Calcium Dysregulation in Post‐ICH Lungs

**FIGURE 2:**
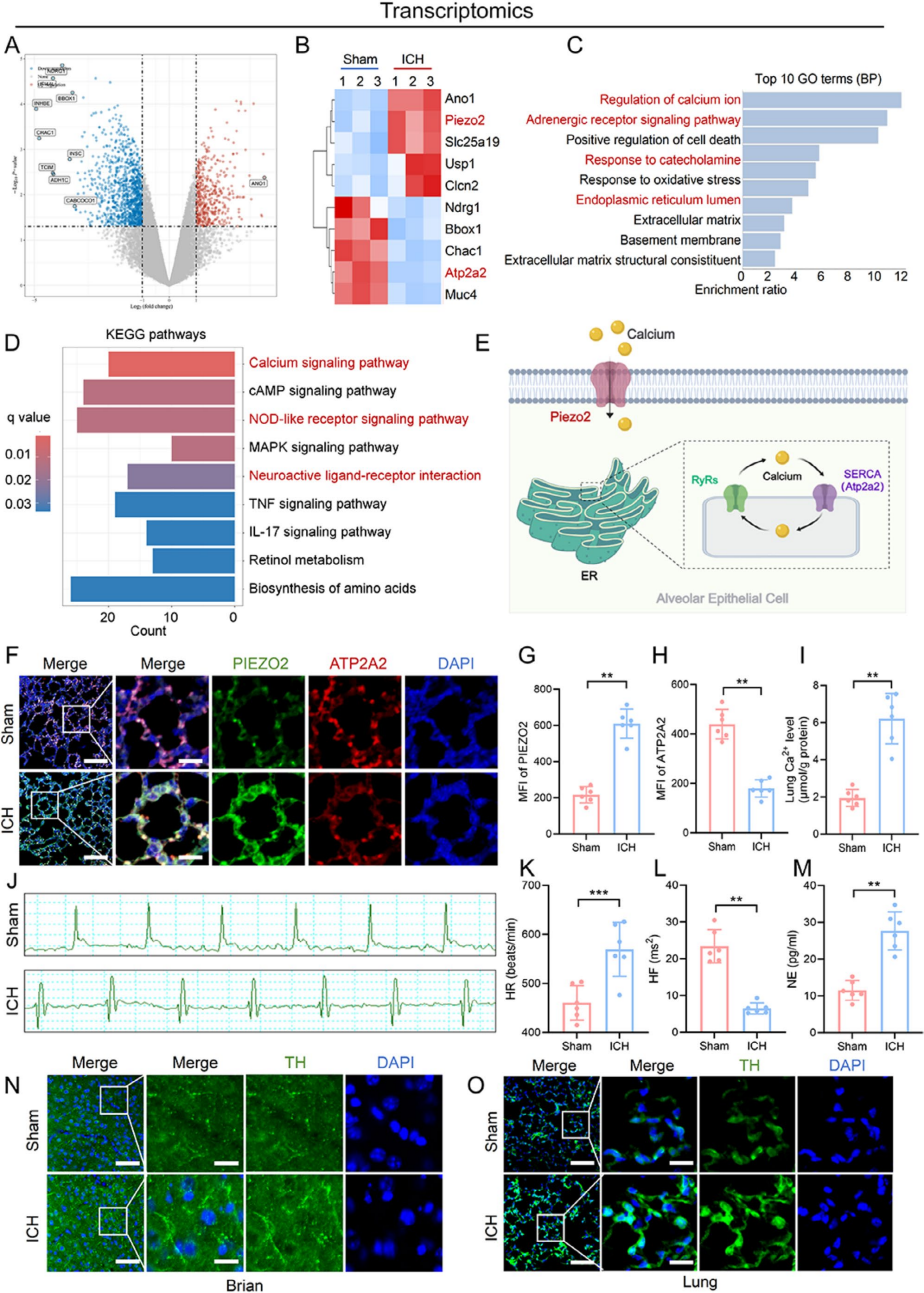
Transcriptomic profiling indicates calcium dyshomeostasis and sympathetic hyperactivation in lung injury secondary to intracerebral hemorrhage (ICH). (A–D) RNA‐sequencing analysis of lung tissues from Sham and ICH mice (day 3). Volcano plot (A), heatmap of top differentially expressed genes (DEGs) (B), and Gene Ontology (C) and KEGG pathway (D) enrichment analyses of DEGs. (E) Schematic illustration of alveolar epithelial calcium transporters highlighting *Piezo2* and *Atp2a2*. (F–H) Representative co‐immunofluorescence (IF) images (F) and mean fluorescence intensity quantification of PIEZO2 (G) and ATP2A2 (H) in lung tissues. Scale bars: 100 μm (overviews) and 20 μm (magnified). (I) Tissue calcium content assay. (J–L) Electrocardiogram monitoring (J) with quantification of heart rate (K) and high‐frequency power (L). (M) Norepinephrine levels in lung tissues. (N, O) Representative IF images of tyrosine hydroxylase in brain (N) and lung (O) sections. Scale bars: 100 μm (overviews) and 20 μm (magnified). Data are mean ± SD. *n* = 6 biologically independent animals per group. Statistical significance was determined by an unpaired two‐tailed Student's *t*‐test (G–I, K–M). *p < 0.05, **p < 0.01 vs. Sham.

To explore the molecular mechanisms of ICH‐induced ALI, we performed transcriptomic sequencing on lung tissues from Sham and ICH‐3d mice, identifying distinct DEGs (Figure 2A,B). GO and KEGG pathway enrichment analyses of these DEGs indicated the involvement of specific pathways. Particularly enriched were the “calcium signaling pathway”, “adrenergic receptor signaling pathway”, and “response to catecholamine” (Figure 2C,D). These findings suggested that ICH‐associated sympathetic activation might contribute to dysregulated calcium homeostasis in the lung.

We further investigated the pulmonary calcium‐handling network. Among the dysregulated calcium‐related genes, the mechanosensitive cation channel PIEZO2 was upregulated, whereas the sarco/endoplasmic reticulum calcium ATPase 2 (ATP2A2), which mediates calcium reuptake, was downregulated (Figure 2E). Immunofluorescence and mRNA analyses confirmed the increased PIEZO2 and decreased ATP2A2 expression in ICH lungs (Figure 2F–H and Figure S2A,B). Correspondingly, the total calcium content in lung tissues was significantly elevated post‐ICH (Figure 2I).

To validate the sympathetic activation implicated by transcriptomics, we performed ECG analysis. ICH mice developed tachycardia and a reduction in the HF component of heart rate variability, indicating enhanced sympathetic tone (Figure 2J–L). This systemic sympathetic activation was supported by increased norepinephrine (NE) levels in the serum, brain, and lung tissues, alongside elevated serum EPI (Figure 2M and Figure S2C–E). Additionally, immunofluorescence for TH showed intensified signals in both brain and lung tissues of ICH mice, suggesting local sympathetic activation (Figure 2N,O). Overall, ICH appears to induce a sympathetic response associated with calcium signaling dysregulation in the injured lung, characterized by PIEZO2 upregulation and ATP2A2 downregulation.

### 3.3 Norepinephrine Promotes Calcium Overload and ER Stress‐Associated Pyroptosis in Alveolar Epithelial Cells

**FIGURE 3:**
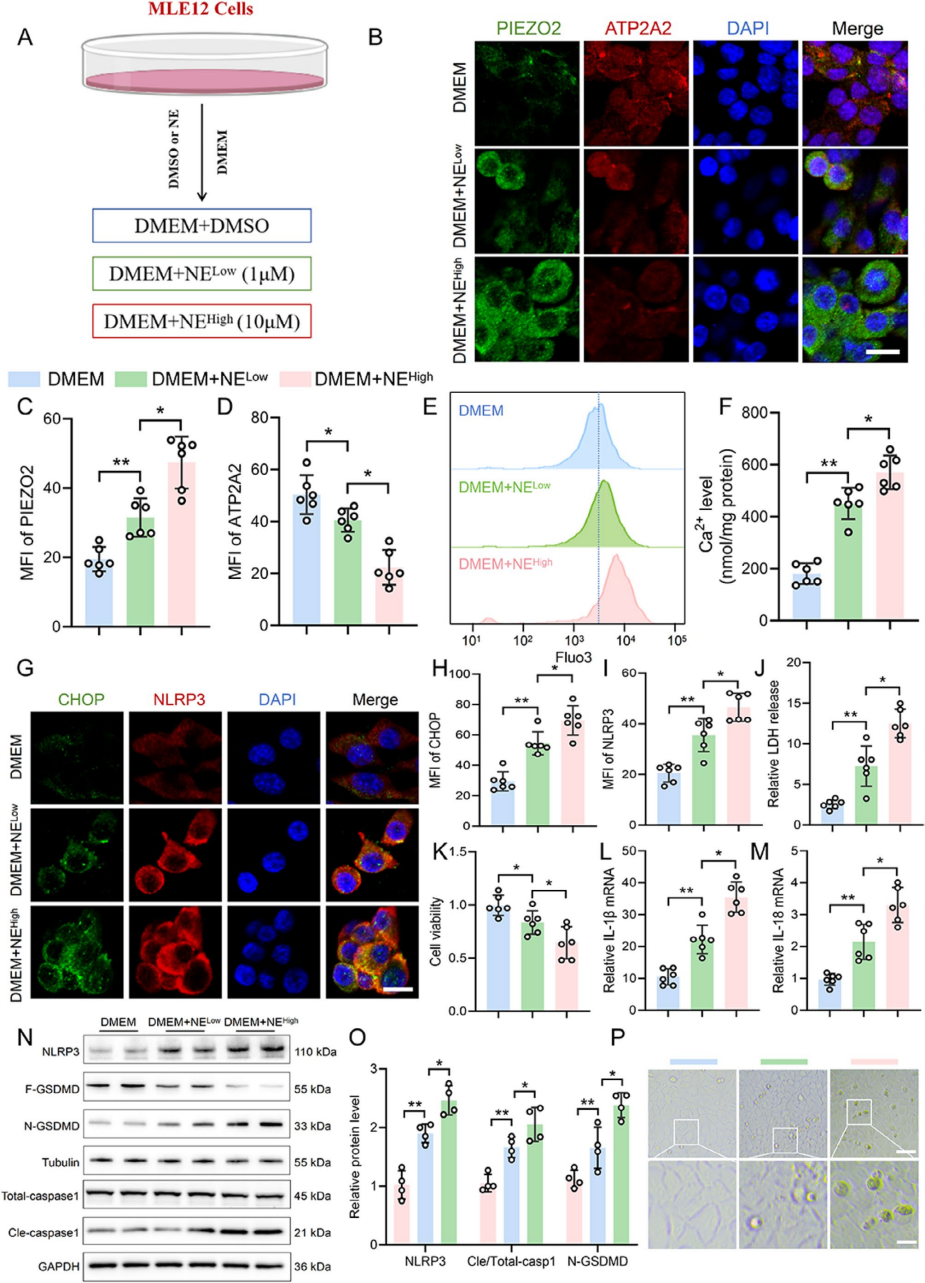
Norepinephrine (NE) promotes alveolar epithelial pyroptosis via PIEZO2/ATP2A2‐associated calcium overload and endoplasmic reticulum (ER) stress in vitro. (A) Schematic of the in vitro experimental design using MLE‐12 cells treated with low (1 μM) or high (10 μM) doses of NE. (B–D) Representative co‐immunofluorescence (IF) images (B) and mean fluorescence intensity (MFI) quantification of PIEZO2 (C) and ATP2A2 (D). Scale bars, 20 μm. (E, F) Intracellular calcium concentration detected by flow cytometry (Fluo‐3) (E) and calcium assay kit (F). (G–I) Representative IF images (G) and MFI quantification of CHOP (H) and NLRP3 (I), indicating the onset of ER stress. Scale bars, 20 μm. (J, K) Cytotoxicity assessed by LDH release (J) and cell viability (K). (L, M) RT‐qPCR analysis of *Il1b* (L) and *Il18* (M) mRNA levels. (N, O) Representative Western blot images (N) and quantification (O) of pyroptosis execution proteins. (P) Representative bright‐field morphology of MLE‐12 cells undergoing pyroptosis. Scale bars: 100 μm and 20 μm. Data are mean ± SD. *n* = 4 (O) or *n* = 6 (C–F, H–M) independent experiments. Statistical significance was determined by one‐way ANOVA with Tukey's *post hoc* test. *p < 0.05, **p < 0.01 between indicated groups.

To investigate the direct effects of sympathetic neurotransmitters on pulmonary pathology, we used the murine alveolar epithelial cell line MLE‐12. Stimulation with NE to mimic the in vivo hyperadrenergic state partially recapitulated the molecular alterations observed in ICH lungs. Exogenous NE treatment dose‐dependently increased PIEZO2 and decreased ATP2A2 expression (Figure 3A–D). These changes in calcium‐handling proteins were accompanied by a dose‐dependent elevation of intracellular calcium concentrations. This elevation was confirmed using Fluo‐3 AM flow cytometry and a calcium assay kit (Figure 3E,F).

We then examined the cellular responses to NE stimulation. Immunofluorescence analysis showed increased expression of CHOP, an ER stress marker, and the inflammasome sensor NLRP3 (Figure 3G–I). These results suggested the activation of ER stress and associated inflammatory pathways. Furthermore, NE treatment appeared to promote cellular pyroptosis. These molecular changes coincided with increased LDH release, reduced cell viability (Figure 3J,K), and elevated mRNA levels of pyroptosis‐related cytokines *Il1b* and *Il18* (Figure 3L,M). Western blotting revealed NLRP3 upregulation and increased cleavage of caspase‐1 and GSDMD (N‐GSDMD) (Figure 3N,O). Morphologically, NE‐treated cells exhibited features consistent with pyroptosis, including cellular ballooning and membrane rupture (Figure 3P). These findings suggest that NE can disrupt calcium homeostasis in alveolar epithelial cells, a process associated with ER stress and NLRP3 inflammasome activation.

### 3.4 VPS35 Knockdown Attenuates Norepinephrine‐Induced Calcium Overload and Pyroptosis in Vitro

**FIGURE 4:**
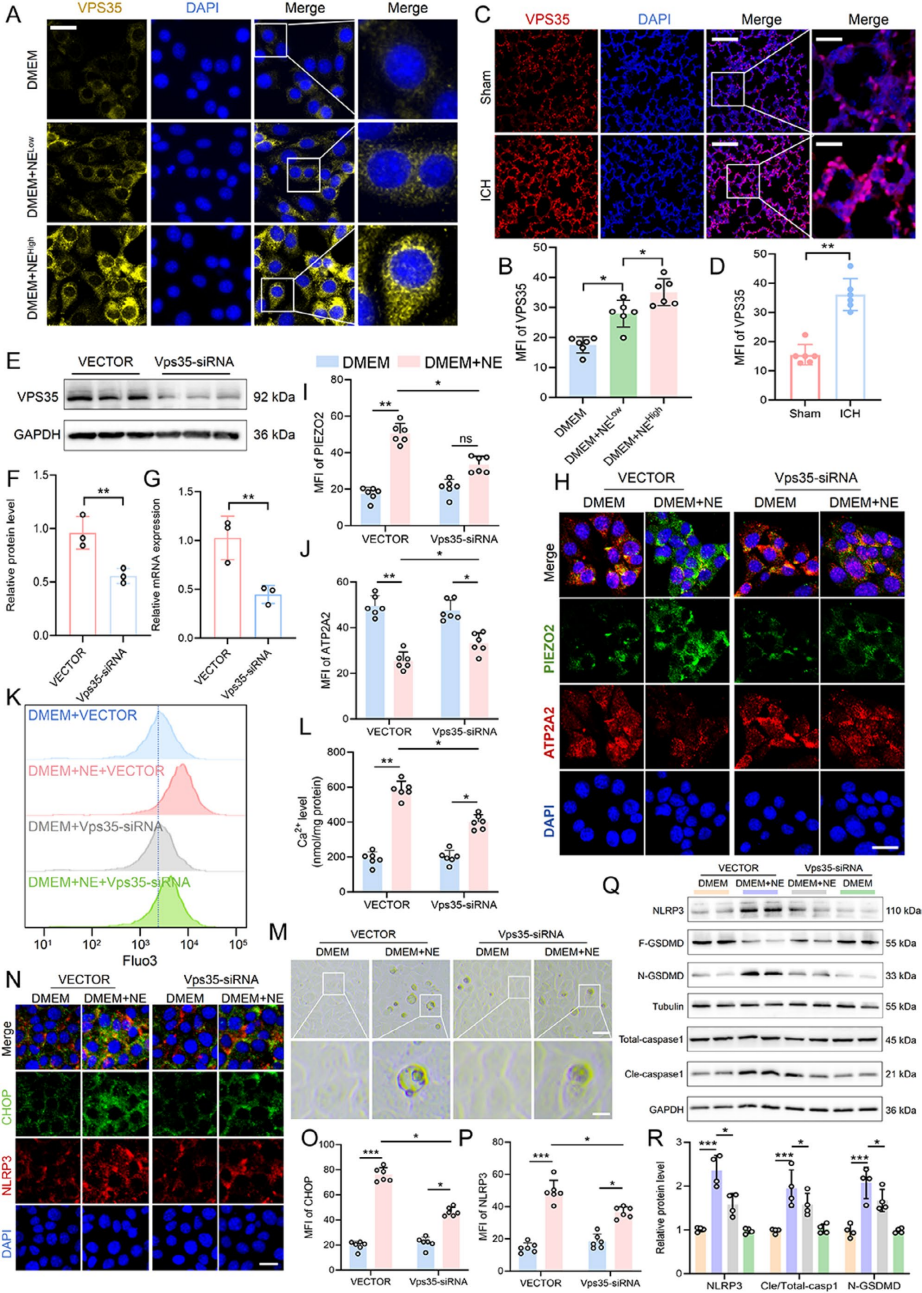
VPS35 upregulation contributes to norepinephrine (NE)‐induced calcium dysregulation and pyroptosis, which are attenuated by *Vps35* silencing. (A–D) Representative immunofluorescence (IF) images and mean fluorescence intensity (MFI) quantification of VPS35 in MLE‐12 cells treated with NE (A, B; scale bars, 50 μm) and in post‐ICH mouse lung tissues (C, D; scale bars, 100 μm and 20 μm). (E–G) Validation of *Vps35* knockdown via siRNA in vitro by Western blot (WB) (E, F) and RT‐qPCR (G). (H–J) co‐immunofluorescence (IF) images (H) and MFI quantification of PIEZO2 (I) and ATP2A2 (J) demonstrating that *Vps35* silencing corrects NE‐induced transporter imbalance. Scale bars, 20 μm. (K, L) Intracellular calcium levels determined by flow cytometry (K) and calcium assay (L). (M) Cellular morphological rescue. Scale bars: 100 μm and 20 μm. (N‐P) IF evaluation of endoplasmic reticulum (ER) stress and pyroptosis markers (CHOP and NLRP3). Scale bars, 20 μm. (Q, R) WB images (Q) and quantification (R) confirming the inhibition of pyroptosis cleavage cascades following *Vps35* knockdown. Data are mean ± SD. *n* = 3 (F, G), *n* = 4 (R), or *n* = 6 (B, D, I–L, O, P) independent samples/animals. Statistical significance was determined by unpaired two‐tailed Student's *t*‐test (D) or one‐way ANOVA with Tukey's *post hoc* test (B, F, G, I–L, O, P, R). *p < 0.05, **p < 0.01, ***p < 0.001 between indicated groups.

To explore the mechanisms of calcium overload in pulmonary epithelial cells, we evaluated the expression profiles of proteins within the intracellular calcium transport network (Figure S3A–D). We observed that the retromer complex component VPS35 exhibited consistent protein upregulation both in vitro following NE stimulation and in vivo in the lungs of ICH mice (Figure 4A–D). To clarify whether this accumulation occurred at the transcriptional or post‐transcriptional level, we assessed the mRNA levels of *Vps35*. RT‐qPCR analysis revealed a significant elevation of *Vps35* mRNA in both the lung tissues of ICH mice and NE‐treated MLE‐12 cells (Figure S3A,B), confirming that NE upregulates VPS35 primarily at the transcriptional level. Subsequently, to investigate its functional role in this pathway, we knocked down VPS35 expression in MLE‐12 cells using specific small interfering RNAs (siRNAs) (Figure 4E–G and Figure S4A–D).

Silencing *Vps35* attenuated the NE‐induced alterations in PIEZO2 and ATP2A2 expression (Figure 4H–J). Consequently, VPS35 knockdown reduced the intracellular calcium overload triggered by NE (Figure 4K,L). We then evaluated the potential cytoprotective effects of VPS35 inhibition. Morphological analysis indicated that VPS35 knockdown alleviated NE‐induced cellular swelling and cell death (Figure 4M). At the molecular level, VPS35 silencing suppressed NE‐induced ER stress and inflammasome activation. This was indicated by reduced fluorescence intensities of CHOP and NLRP3 (Figure 4N–P). Furthermore, the expression levels of pyroptosis markers, including NLRP3, cleaved caspase‐1, and N‐GSDMD, were decreased (Figure 4Q,R). These changes were accompanied by improved cell viability, reduced LDH leakage, and decreased transcription of *Il1b* and *Il18* (Figure S5A–D). Collectively, these findings suggest that VPS35 is involved in bridging sympathetic activation with pulmonary calcium‐associated pyroptosis.

### 3.5 Stellate Ganglion Block Mitigates ICH‐Induced Lung Injury and Improves Cognitive Outcomes

**FIGURE 5:**
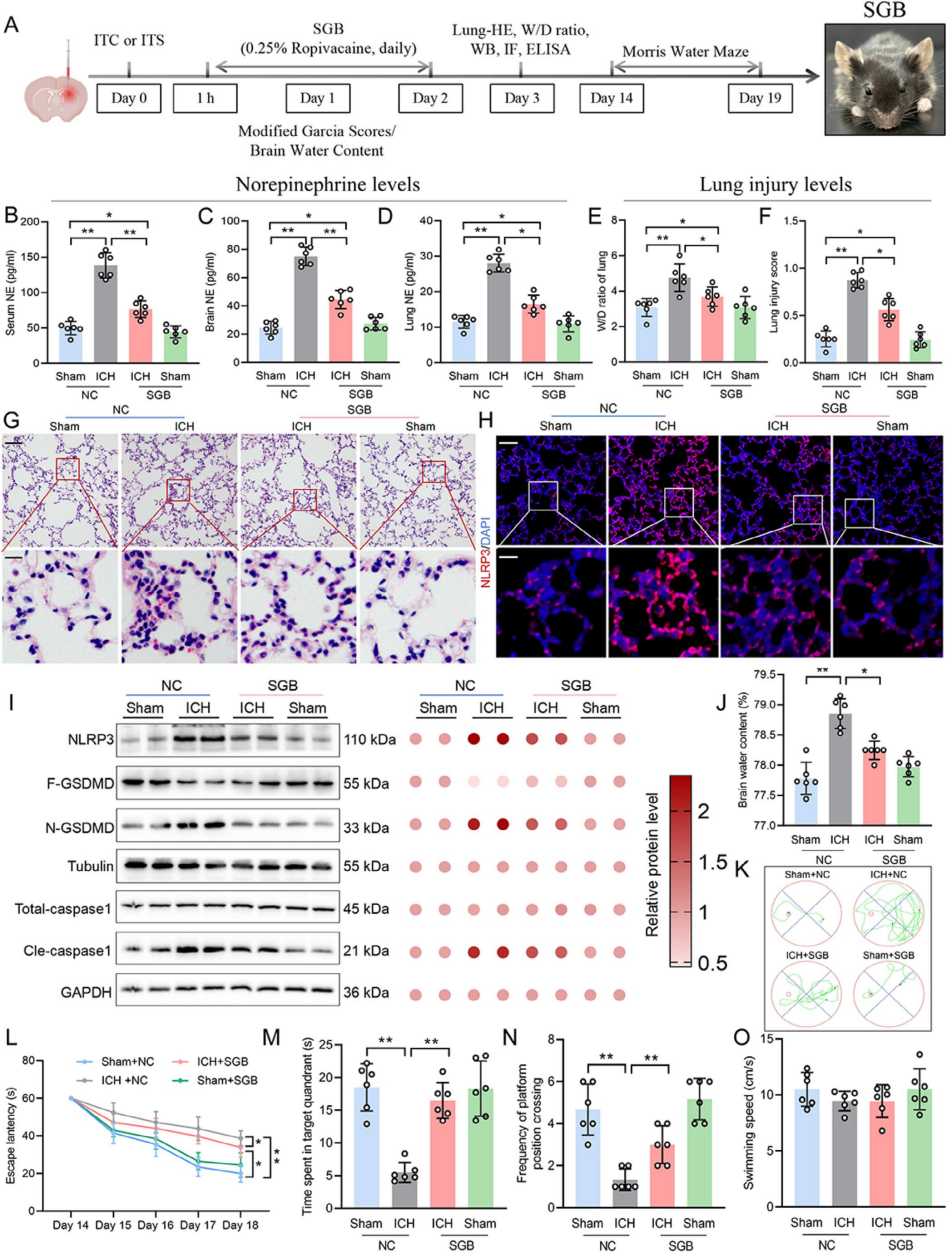
Stellate ganglion block (SGB) mitigates sympathetic overactivation, alveolar pyroptosis, and neurocognitive deficits post‐intracerebral hemorrhage (ICH). (A) Schematic illustrating the SGB interventional strategy in vivo. (B–D) Assessment of norepinephrine levels in serum (B), brain (C), and lung (D), confirming systemic sympathetic blockade. (E–G) Attenuation of pulmonary edema and pathological injury evidenced by lung wet/dry (W/D) ratio (E), injury scores (F), and HE staining (G). (H) Representative immunofluorescence images of NLRP3 in lung tissues. Scale bars: 100 μm and 20 μm. (I) Western blot analysis of pyroptosis‐related proteins (NLRP3, GSDMD‐Full, GSDMD‐N, Total Caspase‐1, and Cleaved Caspase‐1). (J) Quantification of brain water content. (K–O) Morris Water Maze test assessing neurocognitive recovery: Representative trajectories (K), escape latency (L), time in target quadrant (M), platform crossings (N), and swimming speed (O). Data are mean ± SD. *n* = 4 (I) or *n* = 6 (B–F, J, L–O) biologically independent animals. Statistical significance was determined by one‐way ANOVA with Tukey's *post hoc* test (B–F, J, M–O) or two‐way repeated‐measures ANOVA with Bonferroni's *post hoc* test (L). *p < 0.05, **p < 0.01 between indicated groups.

To explore therapeutic strategies targeting the sympathetic‐calcium‐pyroptosis axis, we evaluated two distinct interventions. First, we utilized SGB, a clinical procedure that blocks sympathetic outflow, to modulate the upstream adrenergic response (Figure 5A). SGB intervention significantly reduced NE levels in the serum, brain, and lung tissues of ICH mice (Figure 5B–D). This attenuation of adrenergic drive was associated with altered molecular profiles in the lungs. Specifically, SGB suppressed the ICH‐induced upregulation of VPS35 and PIEZO2, and partially restored ATP2A2 expression (Figure S6A–E).

Functionally, administration of SGB significantly attenuated the ICH‐induced acute lung injury by decreasing the lung W/D ratio (Figure 5E) and improving overall histological architecture (Figure 5F,G). However, while these injury parameters were substantially reduced, they did not fully return to the baseline levels observed in the Sham+NC group, indicating a powerful yet partial protective effect. Furthermore, SGB reduced the activation of the pyroptotic cascade in the alveolar epithelium. This was demonstrated by decreased NLRP3 immunofluorescence (Figure 5H) and the downregulation of pyroptosis‐related proteins, including NLRP3, cleaved caspase‐1, and N‐GSDMD (Figure 5I). Alongside these pulmonary benefits, mitigating the systemic sympathetic activation via SGB also conferred secondary neuroprotective effects. SGB alleviated ICH‐induced brain edema (Figure 5J) and improved spatial learning and memory performance (Figure 5K–O).

### 3.6 Pharmacological Inhibition of PIEZO2 Alleviates ICH‐Induced Pulmonary and Neurological Deficits

**FIGURE 6:**
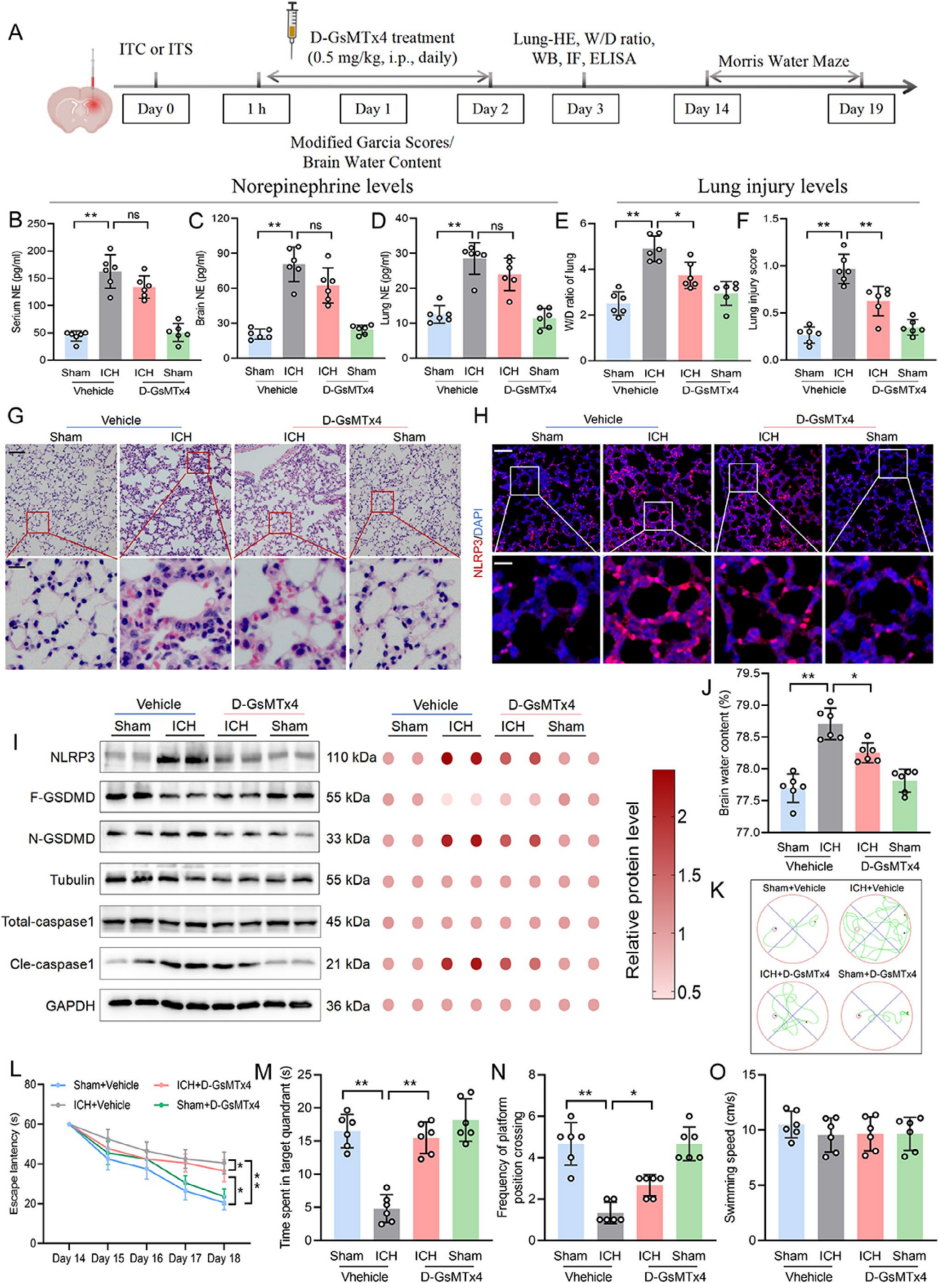
Pharmacological inhibition of PIEZO2 alleviates calcium‐associated pulmonary pyroptosis and improves post‐intracerebral hemorrhage (ICH) outcomes. (A) Schematic of the therapeutic intervention using the PIEZO2 inhibitor, D‐GsMTx4. (B–D) Assessment of norepinephrine concentrations in serum (B), brain (C), and lung (D). (E–G) Evaluation of pulmonary edema and structural damage via wet/dry (W/D) ratio (E), injury scores (F), and HE staining (G). (H) Immunofluorescence images indicating reduced NLRP3 assembly in lungs. Scale bars: 100 μm and 20 μm. (I) Western blot analysis confirming the blunting of the pyroptotic signaling cascade. (J) Brain water content analysis. (K–O) Morris Water Maze behavioral assessment: Trajectories (K), escape latency (L), target quadrant time (M), platform crossings (N), and swimming speed (O). Data are mean ± SD. *n* = 4 (I) or *n* = 6 (B–F, J, L–O) biologically independent animals. Statistical significance was determined by one‐way ANOVA with Tukey's *post hoc* test (B–F, J, M–O) or two‐way repeated‐measures ANOVA with Bonferroni's *post hoc* test (L). *p < 0.05, **p < 0.01 between indicated groups.

To evaluate the specific role of PIEZO2‐mediated calcium overload, we administered the PIEZO2 channel inhibitor D‐GsMTx4 (Figure 6A). Unlike SGB, D‐GsMTx4 treatment did not alter the elevated NE levels in ICH mice, suggesting its effects occur downstream of systemic sympathetic activation (Figure 6B–D). Despite this persistent sympathetic activation, pharmacological inhibition of PIEZO2 attenuated ICH‐induced ALI, significantly reducing pulmonary edema and histological damage (Figure 6E–G).

At the molecular level, D‐GsMTx4 mitigated the altered expression of PIEZO2, VPS35, and ATP2A2 in lung tissues (Figure S7A–E). It also inhibited NLRP3‐associated pyroptosis (Figure 6H,I). Similar to the beneficial effects of SGB, D‐GsMTx4 administration alleviated brain edema and improved cognitive performance post‐ICH (Figure 6J–O). These results suggest that targeted inhibition of PIEZO2, independent of upstream sympathetic modulation, provides therapeutic benefits and may serve as a potential pharmacological approach for ICH‐induced injuries.

## 4 Discussion

This study elucidated molecular mechanisms of ICH‐induced ALI, proposing a “sympathetic‐calcium‐pyroptosis” axis. Mechanistically, we identified VPS35, a retromer complex component, as an upstream regulator in alveolar epithelial cells. Systemic sympathetic overactivation induces VPS35 upregulation, disrupting pulmonary calcium homeostasis via an altered PIEZO2/ATP2A2 expression balance. This leads to intracellular calcium overload, promoting ER stress and NLRP3 inflammasome‐associated pyroptosis. Importantly, targeted interventions at two distinct nodes of this cascade attenuated the brain‐lung crosstalk. Mitigating sympathetic overactivation via SGB, or inhibiting calcium influx with the PIEZO2 blocker D‐GsMTx4, alleviated pulmonary damage and provided secondary neuroprotection.

While ICH‐induced ALI is often attributed to systemic inflammatory cascades [25, 26], these processes typically evolve over hours to days [27, 28, 29, 30]. Our findings suggest sympathetic overactivation as a more rapid initiating event. Acute brain injury triggers centrally mediated sympathetic hyperactivity and elevated catecholamines within minutes to hours [31], known to drive neurogenic pulmonary edema via acute capillary pressure overload [32]. We propose that this neuro‐humoral axis acts as an initial insult, disrupting pulmonary homeostasis and priming the lung for progressive injury, culminating in peak damage around day 3 post‐ICH, likely exacerbated by subsequent inflammation. Clinical evidence supports that mitigating catecholamine effects improves brain injury outcomes [33, 34]. SGB is a recognized clinical method for cervical sympathetic overactivity, demonstrating promise in improving cerebrovascular outcomes by reducing inflammation and oxidative stress [35, 36, 37, 38]. Our study shows SGB intervention at injury peak (day 3) attenuated both pulmonary and secondary cerebral damage, indicating the sympathetic axis remains a therapeutic target throughout disease progression. Thus, SGB offers a translatable strategy to interrupt the brain‐lung pathogenic cycle following ICH.

To elucidate the intracellular events linking sympathetic overactivation to alveolar damage, we investigated the downstream signaling of NE. A key finding of our study is the identification of VPS35 as a potential cellular mediator in this context. Traditionally, VPS35 is recognized as a core component of the retromer complex. It is primarily implicated in endosomal protein sorting and the pathogenesis of neurodegenerative disorders like Parkinson's disease [39, 40]. However, its role in the peripheral respiratory system has remained largely underexplored. Here, we propose a role for VPS35 in contributing to pulmonary calcium dysregulation. Intracellular calcium homeostasis relies on a precise balance between calcium influx and ER‐mediated calcium reuptake [41, 42]. Our data suggest that NE‐induced upregulation of VPS35 disrupts this delicate balance by affecting both the mechanosensitive channel PIEZO2 and the calcium pump ATP2A2 (SERCA2). PIEZO2 is increasingly recognized as a mechanotransducer facilitating calcium influx in pulmonary and epithelial tissues [43]. In our model, elevated VPS35 appears to augment PIEZO2‐mediated calcium entry while concurrently impairing ATP2A2, an essential pump for clearing cytosolic calcium into the ER [44, 45]. The resulting imbalance between calcium influx and clearance induces severe intracellular calcium overload. Such a sustained calcium burden subsequently triggers ER stress, a recognized precursor to inflammatory cell death [46, 47], ultimately promoting pyroptosis in alveolar epithelial cells. By identifying VPS35 as a link between adrenergic activation and calcium‐dependent cell death, our findings expand the known functions of the retromer complex beyond the central nervous system. This offers a new mechanistic perspective on the pathogenesis of acute lung injury.

The intracellular calcium overload driven by the sympathetic‐VPS35 axis likely triggers sustained ER stress in the pulmonary epithelium [48]. Upon exceeding adaptive responses, this stress upregulates the transcription factor CHOP [49, 50]. Our findings suggest that rather than merely inducing apoptosis, CHOP signaling may act as an upstream trigger for the inflammatory cascade. Prolonged ER stress and CHOP activation promote NLRP3 inflammasome assembly, potentially via reactive oxygen species or interaction with TXNIP [51]. This leads to Caspase‐1‐mediated cleavage of GSDMD, driving membrane pore formation and subsequent pyroptosis with pro‐inflammatory cytokine release [52, 53]. Extensive pyroptotic cell death compromises the alveolar‐capillary barrier integrity, facilitating the fluid exudation characteristic of post‐ICH pulmonary edema [54, 55, 56]. Consequently, our findings outline a pathological sequence where an initial adrenergic‐driven calcium influx progresses to GSDMD‐mediated barrier disruption. Together, these data provide a molecular framework for understanding early ICH‐induced acute lung injury.

In addition to unilateral brain‐to‐lung signaling, our study highlights the “brain‐lung‐brain” cycle, a recognized contributor to mortality in neurocritical care [57]. In this context, ICH‐induced acute lung injury exacerbates systemic hypoxia and triggers a systemic inflammatory response. These factors often reflect back to aggravate secondary brain injury [58]. Our data revealed a secondary benefit of localized pulmonary intervention. Directly targeting the lung with the mechanosensitive channel inhibitor D‐GsMTx4 [59] rescued the pulmonary architecture. Furthermore, it attenuated cerebral edema and improved neurocognitive outcomes. This suggests that mitigating PIEZO2‐dependent pulmonary calcium influx helps preserve blood‐gas barrier integrity. This preservation may prevent the hypoxic and inflammatory feedback loop. This supports the concept that protecting the lung can confer secondary protection to the brain. While systemic sympathetic modulation like SGB is effective, it carries potential risks of systemic hypotension [60]. This could compromise cerebral perfusion pressure in acute ICH patients. Therefore, targeting PIEZO2‐mediated calcium influx in the pulmonary epithelium may offer a viable therapeutic approach. Pharmacological blockade with D‐GsMTx4 helps alleviate ALI and potentially attenuates secondary neurological deterioration. This strategy interrupts the pathogenic brain‐lung interaction without altering global sympathetic tone.

It is worth noting that our transcriptomic analysis also identified other critical targets and pathways that warrant attention. For instance, Ano1, which encodes a calcium‐activated chloride channel, was identified as a differentially expressed gene. Furthermore, the cAMP signaling pathway was highly enriched. Given that Ano1 is known to regulate airway secretion and smooth muscle tone, and the cAMP pathway is intricately linked to calcium homeostasis and sympathetic adrenergic signaling, these findings suggest a highly complex molecular network driving lung injury. Although the current study primarily focused on the upstream calcium initiator PIEZO2 and the subsequent ER stress‐pyroptosis axis, we cannot exclude the parallel involvement of the cAMP pathway or downstream Ano1 activation. Investigating the specific roles of Ano1 and cAMP signaling in post‐ICH pulmonary dysfunction represents a promising avenue for our future studies.

While this study provides insights into the pathophysiology of early ICH‐induced lung injury, several limitations exist. First, although pharmacological blockade with D‐GsMTx4 and siRNA‐mediated knockdown were employed, potential off‐target effects cannot be entirely excluded. Future studies using alveolar epithelial‐specific conditional knockout mice for *Vps35* or *Piezo2* would provide more definitive genetic validation. Second, our conclusions are predominantly drawn from murine models of ICH and cultured cell lines, requiring further translational validation. Evaluating the expression profiles of VPS35, PIEZO2, and downstream pyroptotic markers in the BALF or serum from neurocritical care patients is a necessary next step. Third, the present study focused primarily on the vulnerability of alveolar epithelial cells to sympathetic activation. However, the pathogenesis of acute lung injury involves complex intercellular networks. Recent evidence highlights the crosstalk among lung‐resident macrophages, microvascular endothelial cells, and the epithelium in propagating acute alveolar damage [61, 62]. It remains to be determined whether systemic sympathetic activation simultaneously modulates these immune and vascular compartments through comparable mechanisms. Furthermore, it is important to acknowledge that the long‐term cognitive improvements (evaluated by the Morris Water Maze at day 14 post‐ICH) following interventions such as stellate ganglion block (SGB) or D‐GsMTx4 cannot be exclusively attributed to the mitigation of acute lung injury. These systemic interventions may also exert direct neuroprotective effects on the injured brain. Because lung tissue harvesting for histopathological evaluation at the peak of acute lung injury (day 3) precluded long‐term behavioral tracking of the same animals, we were unable to perform an individual‐level correlation analysis between early lung injury severity and late‐stage cognitive outcomes. Future studies incorporating non‐invasive, longitudinal imaging of pulmonary inflammation alongside neurobehavioral assessments in the same cohorts are required to dissect the exact contribution of lung repair to brain recovery. Finally, there are certain limitations regarding the in vitro model used in our study. In the cell experiments, we applied NE at final concentrations of 1 μM and 10 μM in the culture medium to simulate sympathetic overactivation, a dosing strategy consistent with recent in vitro studies [24]. While these concentrations effectively recapitulate cellular stress phenotypes, they are supraphysiologic compared to the circulating plasma NE levels observed in patients or animals (typically in the nanomolar range). Although local NE concentrations at sympathetic nerve endings within the lung tissue might be significantly higher than systemic levels, the use of a high‐dose bolus in our cell culture system may not fully replicate the dynamic and complex microenvironment of in vivo sympathetic surges following ICH. Future studies utilizing co‐culture systems or advanced in vivo localized monitoring are warranted to validate these mechanisms under more physiological conditions. Despite these limitations, our findings outline a potential targetable bidirectional brain‐lung axis. Intercepting this cascade may provide a translational framework for managing ICH‐induced multiorgan dysfunction.

## 5 Conclusions

In conclusion, our study highlights a mechanism underlying ICH‐induced acute lung injury. It suggests that early sympathetic overactivation promotes alveolar epithelial pyroptosis via VPS35‐dependent PIEZO2 activation and subsequent calcium overload. Furthermore, upstream sympathetic modulation using SGB and downstream PIEZO2 inhibition both attenuate pulmonary damage and secondary brain injury. These findings provide preclinical evidence that targeting specific nodes of the brain‐lung crosstalk may serve as a potential therapeutic strategy for managing multiorgan dysfunction in neurocritical care.

## Author Contributions

Shuai Han, Zirui Wang, and Bingchun Yan designed the research; Shuai Han, Zirui Wang, and Qiuyue Zheng collected the data and verified the accuracy of the data. Yinggang Xiao and Zi Wang verified the accuracy of the data; Tianfeng Huang and Feng Guo contributed to data interpretation; Shuai Han and Ju Gao performed the statistical analysis and visualization; Shuai Han and Zirui Wang wrote the manuscript. All authors read, critically reviewed, and approved the final manuscript.

## Funding

This work was supported by the National Natural Science Foundation of China (Grant Nos. 82172190 and 82571398) and the Research Grant of Northern Jiangsu People's Hospital (Grant Nos. SBGP24005 and SBJC23005).

## Ethics Statement

All animal experimental procedures and protocols in this study were reviewed and rigorously approved by the Institutional Animal Care and Use Committee (IACUC) of Yangzhou University School of Medicine (Approval ID: No. 20250315). All procedures were conducted in strict accordance with the institutional guidelines for the care and use of laboratory animals to minimize suffering.

## Consent

The authors have nothing to report.

## Conflicts of Interest

The authors declare no conflicts of interest.

## Supporting information


**Figure S1:** Extended evaluation of pulmonary edema, barrier dysfunction, and functional decline following intracerebral hemorrhage (ICH). (A, B) Time‐course evaluation of bronchoalveolar lavage fluid protein concentration (A) and lung wet/dry (W/D) ratio (B). (C–E) Serum ELISA quantification of TNF‐α (C), IL‐6 (D), and IL‐1β (E). (F) Gross morphology of unperfused lungs (top) and Evans blue extravasation (bottom) on day 3 post‐ICH. (G) Immunofluorescence staining of tight junction proteins (ZO‐1 and Claudin‐5) demonstrating severe barrier disruption on day 3. Scale bar, 100 μm. (H) Assessment of pulmonary function parameters. Data are mean ± SD. *n* = 6 independent animals. Statistical significance was determined by unpaired two‐tailed Student's *t*‐test (C–E) or one‐way ANOVA with Tukey's *post hoc* test (A, B). *p < 0.05, **p < 0.01, ***p < 0.001, ****p < 0.0001 vs. Sham; ns, not significant.
**Figure S2:** Systemic catecholamine surge and transcriptional alterations of calcium transporters post‐intracerebral hemorrhage (ICH). (A, B) RT‐qPCR analysis demonstrating the upregulation of Piezo2 (A) and Atp2a2 (B) mRNA in lung tissues post‐ICH. (C–E) ELISA quantification of serum Epinephrine (EPI) (C), serum NE (D), and lung EPI (E). Data are mean ± SD. *n* = 6 independent animals. Statistical significance was determined by an unpaired two‐tailed Student's t‐test. *p < 0.05, ***p < 0.001 vs. Sham; ns, not significant.
**Figure S3:** Sympathetic signaling inhibited calcium handling proteins. (A, B) Validation of mRNA expression levels of 10 calcium transport‐related proteins in MLE‐12 cells (A) and mouse lung tissues (B). (C–F) Representative immunofluorescence images and mean fluorescence intensity quantification of STIM1 in vitro (C, D; scale bar, 20 μm) and in vivo (E, F; scale bar, 50 μm), showing no significant differences. Data are mean ± SD. *n* = 3 independent samples/animals. Statistical significance was determined by one‐way ANOVA with Tukey's post hoc test (D) or unpaired two‐tailed Student's t‐test (F). *p < 0.05, **p < 0.01 between indicated groups; ns, not significant.
**Figure S4:** Validation of Vps35 knockdown efficiency in MLE‐12 cells. (A) Representative bright‐field (top) and GFP fluorescence (bottom) images of MLE‐12 cells 24 h post‐transfection. Scale bar, 200 μm. (B, C) WB images (B) and densitometric quantification (C) of VPS35 protein levels after transfection with scrambled or three distinct Vps35‐siRNAs. (D) RT‐qPCR validation of Vps35 mRNA transcription levels. Data are mean ± SD. *n* = 3 independent cell cultures. Statistical significance was determined by one‐way ANOVA with Tukey's post hoc test. **p < 0.01, ***p < 0.001 vs. scr‐siRNA.
**Figure S5:** Vps35 knockdown alleviates norepinephrine (NE)‐induced cytotoxicity and pro‐inflammatory cytokine transcription. (A, B) Cytotoxicity and cell survival assessed via LDH release (A) and viability assay (B) in cells transfected with control vector or Vps35‐siRNA, with or without NE. (C, D) RT‐qPCR analysis of downstream pyroptosis‐related pro‐inflammatory cytokines Il1b (C) and Il18 (D). Data are mean ± SD. *n* = 6 independent cell cultures. Statistical significance was determined by one‐way ANOVA with Tukey's post hoc test. *p < 0.05, ***p < 0.001 between indicated groups.
**Figure S6:** Stellate ganglion block (SGB) reverses the intracerebral hemorrhage (ICH)‐induced molecular dysregulation of PIEZO2, VPS35, and ATP2A2 in the lung. (A, B) Representative Western Blot images (A) and quantification (B) of PIEZO2, VPS35, and ATP2A2 in lung homogenates. (C–E) Representative co‐immunofluorescence images of PIEZO2 (green) and ATP2A2 (red) (C) and corresponding mean fluorescence intensity quantification (D, E) among simplified experimental groups. Data are mean ± SD. *n* = 4 (B) or *n* = 6 (D, E) biologically independent animals. Statistical significance was determined by one‐way ANOVA with Tukey's post hoc test. *p < 0.05, **p < 0.01 between indicated groups.
**Figure S7:** D‐GsMTx4 treatment restores the expression profiles of VPS35 and ATP2A2 post‐intracerebral hemorrhage (ICH). (A, B) Immunoblotting analysis (A) and quantitative densitometry (B) of PIEZO2, VPS35, and ATP2A2 in lung tissues. (C–E) Representative co‐immunofluorescence images (C) and mean fluorescence intensity quantification of PIEZO2 (D) and ATP2A2 (E), indicating that specific PIEZO2 inhibition reciprocally rescues VPS35 and ATP2A2 alterations. Data are mean ± SD. *n* = 4 (B) or *n* = 6 (D, E) biologically independent animals. Statistical significance was determined by one‐way ANOVA with Tukey's post hoc test. *p < 0.05, **p < 0.01, ***p < 0.001 between indicated groups.

**Table S1:** Oligonucleotide sequences of small interfering RNAs (siRNAs) for mouse *Vps35* silencing.
**Table S2:** List of primer sequences.

## Data Availability

The data that support the findings of this study are available on request from the corresponding author. The data are not publicly available due to privacy or ethical restrictions.

## References

[1] M. Ziaka and A. Exadaktylos , “Brain‐Lung Interactions and Mechanical Ventilation in Patients With Isolated Brain Injury,” Critical Care 25 (2021): 358. 34645485 10.1186/s13054-021-03778-0 PMC8512596

[2] X. Li , J. Deng , Y. Long , et al., “Focus on Brain‐Lung Crosstalk: Preventing or Treating the Pathological Vicious Circle Between the Brain and the Lung,” Neurochemistry International 178 (2024): 105768. 38768685 10.1016/j.neuint.2024.105768

[3] P. Wang , L. Jin , M. Zhang , et al., “Blood‐Brain Barrier Injury and Neuroinflammation Induced by SARS‐CoV‐2 in a Lung‐Brain Microphysiological System,” Nature Biomedical Engineering 8 (2024): 1053–1068. 10.1038/s41551-023-01054-w 37349391

[4] N. H. Johnson , N. G. Casanova , S. P. White , et al., “Lung‐Brain Axis‐Generated Inflammatory Biomarkers in Traumatic Brain Injury and Acute Respiratory Distress Syndrome: Role of Mechanical Ventilation/Stress,” Advances in Biomarker Sciences and Technology 7 (2025): 238–247. 41497388 10.1016/j.abst.2025.08.002 PMC12768522

[5] C. Cordonnier , A. Demchuk , W. Ziai , and C. S. Anderson , “Intracerebral Haemorrhage: Current Approaches to Acute Management,” Lancet 392 (2018): 1257–1268. 30319113 10.1016/S0140-6736(18)31878-6

[6] Y. Liu , F. Li , L. Tang , et al., “Extracellular Mitochondria Contribute to Acute Lung Injury via Disrupting Macrophages After Traumatic Brain Injury,” Journal of Neuroinflammation 22 (2025): 63. 40038717 10.1186/s12974-025-03390-x PMC11881407

[7] J. Zhao , N.‐X. Xuan , W. Cui , and B.‐P. Tian , “Neurogenic Pulmonary Edema Following Acute Stroke: The Progress and Perspective,” Biomedicine & Pharmacotherapy 130 (2020): 110478. 32739737 10.1016/j.biopha.2020.110478

[8] K. M. Busl and T. P. Bleck , “Neurogenic Pulmonary Edema,” Critical Care Medicine 43 (2015): 1710–1715. 26066018 10.1097/CCM.0000000000001101

[9] M. Xue and V. W. Yong , “Neuroinflammation in Intracerebral Haemorrhage: Immunotherapies With Potential for Translation,” Lancet Neurology 19 (2020): 1023–1032. 33212054 10.1016/S1474-4422(20)30364-1

[10] P. J. Winklewski , M. Radkowski , and U. Demkow , “Cross‐Talk Between the Inflammatory Response, Sympathetic Activation and Pulmonary Infection in the Ischemic Stroke,” Journal of Neuroinflammation 11 (2014): 213. 25539803 10.1186/s12974-014-0213-4 PMC4297381

[11] S. Naredi , G. Lambert , E. Edén , et al., “Increased Sympathetic Nervous Activity in Patients With Nontraumatic Subarachnoid Hemorrhage,” Stroke 31 (2000): 901–906. 10753996 10.1161/01.str.31.4.901

[12] C. Li , W. Chen , F. Lin , et al., “Functional Two‐Way Crosstalk Between Brain and Lung: The Brain‐Lung Axis,” Cellular and Molecular Neurobiology 43 (2023): 991–1003. 35678887 10.1007/s10571-022-01238-z PMC9178545

[13] M. Szczot , J. Liljencrantz , N. Ghitani , et al., “PIEZO2 Mediates Injury‐Induced Tactile Pain in Mice and Humans,” Science Translational Medicine 10 (2018): eaat9892. 30305456 10.1126/scitranslmed.aat9892 PMC6875774

[14] M. Szczot , A. R. Nickolls , R. M. Lam , and A. T. Chesler , “The Form and Function of PIEZO2,” Annual Review of Biochemistry 90 (2021): 507–534. 10.1146/annurev-biochem-081720-023244 PMC8794004 34153212

[15] B. Coste , J. Mathur , M. Schmidt , et al., “Piezo1 and Piezo2 Are Essential Components of Distinct Mechanically Activated Cation Channels,” Science 330 (2010): 55–60. 20813920 10.1126/science.1193270 PMC3062430

[16] M.‐M. Zhao , T.‐T. Ren , J.‐K. Wang , et al., “Endoplasmic Reticulum Membrane Remodeling by Targeting Reticulon‐4 Induces Pyroptosis to Facilitate Antitumor Immune,” Protein & Cell 16 (2025): 121–135. 39252612 10.1093/procel/pwae049 PMC11786723

[17] S. Mrozek , J.‐M. Constantin , and T. Geeraerts , “Brain‐Lung Crosstalk: Implications for Neurocritical Care Patients,” World Journal of Critical Care Medicine 4 (2015): 163–178. 26261769 10.5492/wjccm.v4.i3.163 PMC4524814

[18] S. Mrozek , J. Gobin , J.‐M. Constantin , O. Fourcade , and T. Geeraerts , “Crosstalk Between Brain, Lung and Heart in Critical Care,” Anaesthesia Critical Care & Pain Medicine 39 (2020): 519–530. 10.1016/j.accpm.2020.06.016 32659457

[19] S. Chen , J. Peng , P. Sherchan , et al., “TREM2 Activation Attenuates Neuroinflammation and Neuronal Apoptosis via PI3K/Akt Pathway After Intracerebral Hemorrhage in Mice,” Journal of Neuroinflammation 17 (2020): 168. 32466767 10.1186/s12974-020-01853-x PMC7257134

[20] Z.‐M. Shi , J.‐J. Jing , Z.‐J. Xue , et al., “Stellate Ganglion Block Ameliorated Central Post‐Stroke Pain With Comorbid Anxiety and Depression Through Inhibiting HIF‐1α/NLRP3 Signaling Following Thalamic Hemorrhagic Stroke,” Journal of Neuroinflammation 20 (2023): 82. 36944982 10.1186/s12974-023-02765-2 PMC10031944

[21] H. He , J. Zhou , S. Cao , W. Liu , Z. Mei , and M. Liu , “Electroacupuncture Attenuates Intestinal Epithelial Ferroptosis in Inflammatory Bowel Disease via Piezo1‐Mediated Mitochondrial Homeostasis,” Chinese Medicine 20, no. 1 (2025): 161. 41047414 10.1186/s13020-025-01218-7 PMC12498448

[22] Y. Xiao , Y. Zhang , W. Yuan , C. Wang , T. Huang , and J. Gao , “Piezo2 Contributes to Traumatic Brain Injury by Activating the RhoA/ROCK1 Pathways,” Molecular Neurobiology 61, no. 10 (2024): 7419–7430. 38388773 10.1007/s12035-024-04058-y PMC11415480

[23] W. Cai , D. Xu , C. Zeng , et al., “Modulating Lysine Crotonylation in Cardiomyocytes Improves Myocardial Outcomes,” Circulation Research 131 (2022): 456–472. 35920168 10.1161/CIRCRESAHA.122.321054

[24] N. Chen , L. Guo , L. Wang , S. Dai , X. Zhu , and E. Wang , “Sleep Fragmentation Exacerbates Myocardial Ischemia–Reperfusion Injury by Promoting Copper Overload in Cardiomyocytes,” Nature Communications 15, no. 1 (2024): 3834. 10.1038/s41467-024-48227-y PMC11076509 38714741

[25] N. Villalba , Y. Ma , S. A. Gahan , et al., “Lung Infection by *Pseudomonas aeruginosa* Induces Neuroinflammation and Blood‐Brain Barrier Dysfunction in Mice,” Journal of Neuroinflammation 20 (2023): 127. 37245027 10.1186/s12974-023-02817-7 PMC10223932

[26] M. Witzenrath and W. M. Kuebler , “The Lung‐Brain Axis in Ventilator‐Induced Brain Injury: Enter IL‐6,” American Journal of Respiratory Cell and Molecular Biology 65 (2021): 339–340. 34153209 10.1165/rcmb.2021-0233ED PMC8525199

[27] B. Hei , J. Ouyang , J. Zhou , D. Wang , Z. Miao , and R.‐E. Liu , “Raddeanin A (RA) Reduced Acute Inflammatory Injury in Mouse Experimental Cerebral Hemorrhage by Suppression of TLR4,” International Journal of Medical Sciences 19 (2022): 1235–1240. 35928716 10.7150/ijms.73007 PMC9346382

[28] P.‐T. Chiang , L.‐K. Tsai , and H.‐H. Tsai , “New Targets in Spontaneous Intracerebral Hemorrhage,” Current Opinion in Neurology 38 (2025): 10–17. 39325041 10.1097/WCO.0000000000001325 PMC11706352

[29] F. Kibria , O. A. Bragina , A. O. Trofimov , and D. Bragin , “Bidirectional lnterplay Between Traumatic Brain Injury and Cardiovascular Dysfunction in Athletes,” Journal of Clinical Medicine 14 (2025): 7712. 41227109 10.3390/jcm14217712 PMC12609428

[30] L. Li and S. B. Murthy , “Cardiovascular Events After Intracerebral Hemorrhage,” Stroke 53 (2022): 2131–2141. 35674043 10.1161/STROKEAHA.122.036884 PMC9247019

[31] Y. Hasegawa , H. Uchikawa , S. Kajiwara , and M. Morioka , “Central Sympathetic Nerve Activation in Subarachnoid Hemorrhage,” Journal of Neurochemistry 160 (2022): 34–50. 34525222 10.1111/jnc.15511

[32] Y. Nakata , Y. Takasaki , H. Nandate , et al., “Pediatric Neurogenic Pulmonary Edema After Brain Tumor Removal Complicated With Severe Myocardial Injury: A Case Report,” American Journal of Case Reports 25 (2024): e943645. 38711258 10.12659/AJCR.943645 PMC11087668

[33] S. Hart , M. Lannon , A. Chen , A. Martyniuk , S. Sharma , and P. T. Engels , “Beta Blockers in Traumatic Brain Injury: A Systematic Review and Meta‐Analysis,” Trauma Surgery & Acute Care Open 8 (2023): e001051. 36895782 10.1136/tsaco-2022-001051 PMC9990673

[34] I. Zagales , S. Selvakumar , M. Ngatuvai , et al., “Beta‐Blocker Therapy in Patients With Severe Traumatic Brain Injury: A Systematic Review and Meta‐Analysis,” American Surgeon 89 (2023): 2020–2029. 35575287 10.1177/00031348221101583

[35] Y. Nie , R. Song , W. Chen , Z. Qin , J. Zhang , and J. Tang , “Effects of Stellate Ganglion Block on Cerebrovascular Vasodilation in Elderly Patients and Patients With Subarachnoid Haemorrhage,” British Journal of Anaesthesia 117 (2016): 131–132. 27317713 10.1093/bja/aew157 PMC4913408

[36] N. Hu , Y. Wu , B.‐Z. Chen , J.‐F. Han , and M.‐T. Zhou , “Protective Effect of Stellate Ganglion Block on Delayed Cerebral Vasospasm in an Experimental Rat Model of Subarachnoid Hemorrhage,” Brain Research 1585 (2014): 63–71. 25128600 10.1016/j.brainres.2014.08.012

[37] F. Chouairi , M. Fudim , A. Benak , et al., “Factors Associated With Stellate Ganglion Block Success in Recurrent Ventricular Arrhythmias,” ESC Heart Failure 12 (2025): 110–117. 39253899 10.1002/ehf2.15072 PMC11769651

[38] R. A. Patel , J. M. Condrey , R. M. George , B. J. Wolf , and S. H. Wilson , “Stellate Ganglion Block Catheters for Refractory Electrical Storm: A Retrospective Cohort and Care Pathway,” Regional Anesthesia and Pain Medicine 48 (2023): 224–228. 36725213 10.1136/rapm-2022-104172 PMC10251217

[39] D. R. Alessi , P. J. Cullen , M. Cookson , K. M. Merchant , and S. A. Small , “Retromer‐Dependent Lysosomal Stress in Parkinson's Disease,” Philosophical Transactions of the Royal Society of London. Series B, Biological Sciences 379 (2024): 20220376. 38368937 10.1098/rstb.2022.0376 PMC10874697

[40] J. Rowlands and D. J. Moore , “VPS35 and Retromer Dysfunction in Parkinson's Disease,” Philosophical Transactions of the Royal Society of London. Series B, Biological Sciences 379 (2024): 20220384. 38368930 10.1098/rstb.2022.0384 PMC10874700

[41] S. Marchi , M. Bittremieux , S. Missiroli , et al., “Endoplasmic Reticulum‐Mitochondria Communication Through Ca2+ Signaling: The Importance of Mitochondria‐Associated Membranes (MAMs),” Advances in Experimental Medicine and Biology 997 (2017): 49–67. 28815521 10.1007/978-981-10-4567-7_4

[42] M. J. Berridge , “The Inositol Trisphosphate/Calcium Signaling Pathway in Health and Disease,” Physiological Reviews 96 (2016): 1261–1296. 27512009 10.1152/physrev.00006.2016

[43] M. Zheng , N. A. Borkar , Y. Yao , et al., “Mechanosensitive Channels in Lung Disease,” Frontiers in Physiology 14 (2023): 1302631. 38033335 10.3389/fphys.2023.1302631 PMC10684786

[44] F. Gonnot , L. Boulogne , C. Brun , et al., “SERCA2 Phosphorylation at Serine 663 Is a Key Regulator of Ca2+ Homeostasis in Heart Diseases,” Nature Communications 14 (2023): 3346. 10.1038/s41467-023-39027-x PMC10250397 37291092

[45] Y. Shiga , A. G. Rangel Olguin , S. El Hajji , et al., “Endoplasmic Reticulum Stress‐Related Deficits in Calcium Clearance Promote Neuronal Dysfunction That Is Prevented by SERCA2 Gene Augmentation,” Cell Reports Medicine 5 (2024): 101839. 39615485 10.1016/j.xcrm.2024.101839 PMC11722116

[46] I. Tabas and D. Ron , “Integrating the Mechanisms of Apoptosis Induced by Endoplasmic Reticulum Stress,” Nature Cell Biology 13 (2011): 184–190. 21364565 10.1038/ncb0311-184 PMC3107571

[47] Z. Sun , W. He , H. Meng , P. Li , and J. Qu , “Endoplasmic Reticulum Stress in Acute Lung Injury and Pulmonary Fibrosis,” FASEB Journal 38 (2024): e70232. 39651914 10.1096/fj.202401849RR

[48] S. Zhang , Y. Lv , X. Luo , et al., “Homocysteine Promotes Atherosclerosis Through Macrophage Pyroptosis via Endoplasmic Reticulum Stress and Calcium Disorder,” Molecular Medicine 29 (2023): 73. 37308812 10.1186/s10020-023-00656-z PMC10262416

[49] Q. Hong , Y. Zhang , W. Lin , et al., “Negative Feedback of the cAMP/PKA Pathway Regulates the Effects of Endoplasmic Reticulum Stress‐Induced NLRP3 Inflammasome Activation on Type II Alveolar Epithelial Cell Pyroptosis as a Novel Mechanism of BLM‐Induced Pulmonary Fibrosis,” Journal of Immunology Research 2022 (2022): 2291877. 36033388 10.1155/2022/2291877 PMC9410862

[50] Q. Yuan , R. Zhang , J. Hu , et al., “Endoplasmic Reticulum Stress‐Induced CHOP Activation Mediates NLRP3 Inflammasome‐Dependent Pyroptosis in BDE‐47‐Induced Cognitive Dysfunction,” Neurotoxicology 113 (2026): 103409. 41713751 10.1016/j.neuro.2026.103409

[51] N.‐N. Wang , X.‐X. Zhang , P. Shen , et al., “Pinelliae Rhizoma Alleviated Acute Lung Injury Induced by Lipopolysaccharide via Suppressing Endoplasmic Reticulum Stress‐Mediated NLRP3 Inflammasome,” Frontiers in Pharmacology 13 (2022): 883865. 36046826 10.3389/fphar.2022.883865 PMC9421150

[52] T. Jiang , E. Liu , Z. Li , et al., “SIRT1‐Rab7 Axis Attenuates NLRP3 and STING Activation Through Late Endosomal‐Dependent Mitophagy During Sepsis‐Induced Acute Lung Injury,” International Journal of Surgery 110 (2024): 2649–2668. 38445453 10.1097/JS9.0000000000001215 PMC11093444

[53] W.‐J. Zhong , T. Liu , H.‐H. Yang , et al., “TREM‐1 Governs NLRP3 Inflammasome Activation of Macrophages by Firing Up Glycolysis in Acute Lung Injury,” International Journal of Biological Sciences 19 (2023): 242–257. 36594089 10.7150/ijbs.77304 PMC9760435

[54] K. M. Busl , E. G. Bogossian , J. Claassen , et al., “Beyond the Bleed: Complications After Aneurysmal Subarachnoid Hemorrhage. Pathophysiology, Clinical Implications, and Management Strategies: A Review,” Critical Care 29 (2025): 414. 41029753 10.1186/s13054-025-05640-z PMC12895635

[55] M. Shan , S. Zhang , Z. Luo , et al., “Itaconate Promotes Inflammatory Responses in Tissue‐Resident Alveolar Macrophages and Exacerbates Acute Lung Injury,” Cell Metabolism 37 (2025): 1750–1765.e7. 40527316 10.1016/j.cmet.2025.05.012

[56] H. Liu , J. Dong , C. Xu , et al., “Acute Lung Injury: Pathogenesis and Treatment,” Journal of Translational Medicine 23 (2025): 926. 40826415 10.1186/s12967-025-06994-2 PMC12363007

[57] M. Ziaka and A. Exadaktylos , “Pathophysiology of Acute Lung Injury in Patients With Acute Brain Injury: The Triple‐Hit Hypothesis,” Critical Care 28 (2024): 71. 38454447 10.1186/s13054-024-04855-w PMC10918982

[58] I. Domínguez de la Cruz , L. Blanch , and G. M. Albaiceta , “Little Big Issues in Brain‐Lung Crosstalk,” American Journal of Respiratory and Critical Care Medicine 211 (2025): 1317–1318. 10.1164/rccm.202503-0701LE PMC12264699 40344671

[59] C. Alcaino , K. Knutson , P. A. Gottlieb , G. Farrugia , and A. Beyder , “Mechanosensitive Ion Channel Piezo2 Is Inhibited by D‐GsMTx4,” Channels 11 (2017): 245–253. 28085630 10.1080/19336950.2017.1279370 PMC5463886

[60] V. Goel , A. M. Patwardhan , M. Ibrahim , C. L. Howe , D. M. Schultz , and H. Shankar , “Complications Associated With Stellate Ganglion Nerve Block: A Systematic Review,” Regional Anesthesia and Pain Medicine 44 (2019): 669–679. 10.1136/rapm-2018-100127 PMC9034660 30992414

[61] Z. Zhu , Y. Zhang , H. Chen , and H. Zhang , “Cell‐Cell Crosstalk in the Pathogenesis of Acute Lung Injury and Acute Respiratory Distress Syndrome,” Tissue Barriers 13 (2025): 2452082. 39798076 10.1080/21688370.2025.2452082 PMC12506906

[62] S. Osorio‐Valencia and B. Zhou , “Roles of Macrophages and Endothelial Cells and Their Crosstalk in Acute Lung Injury,” Biomedicine 12 (2024): 632. 10.3390/biomedicines12030632 PMC10968255 38540245

