# Supplementary material for: Sympathetic Overactivation Drives Neurogenic Alveolar Epithelial Pyroptosis via the PIEZO2‐ER Stress Pathway in Acute Lung Injury Following Intracerebral Hemorrhage
Source: CNS Neurosci Ther. 2026 Jul 6;32(7):e71010. doi: 10.1002/cns.71010 (PMC13334374; doi:10.1002/cns.71010)
Supplement: Supplementary file 1 — Figure S1: Extended evaluation of pulmonary edema, barrier dysfunction, and functional decline following intracerebral hemorrhage (ICH). (A, B) Time‐course evaluation of bronchoalveolar lavage fluid protein concentration (A) and lung wet/dry (W/D) ratio (B). (C–E) Serum ELISA quantification of TNF‐α (C), IL‐6 (D), and IL‐1β (E). (F) Gross morphology of unperfused lungs (top) and Evans blue extravasation (bottom) on day 3 post‐ICH. (G) Immunofluorescence staining of tight junction proteins (ZO‐1 and Claudin‐5) demonstrating severe barrier disruption on day 3. Scale bar, 100 μm. (H) Assessment of pulmonary function parameters. Data are mean ± SD. n = 6 independent animals. Statistical significance was determined by unpaired two‐tailed Student's t‐test (C–E) or one‐way ANOVA with Tukey's post hoc test (A, B). *p < 0.05, **p < 0.01, ***p < 0.001, ****p < 0.0001 vs. Sham; ns, not significant. Figure S2: Systemic catecholamine surge and transcriptional alterations of calcium transporters post‐intracerebral hemorrhage (ICH). (A, B) RT‐qPCR analysis demonstrating the upregulation of Piezo2 (A) and Atp2a2 (B) mRNA in lung tissues post‐ICH. (C–E) ELISA quantification of serum Epinephrine (EPI) (C), serum NE (D), and lung EPI (E). Data are mean ± SD. n = 6 independent animals. Statistical significance was determined by an unpaired two‐tailed Student's t‐test. *p < 0.05, ***p < 0.001 vs. Sham; ns, not significant. Figure S3: Sympathetic signaling inhibited calcium handling proteins. (A, B) Validation of mRNA expression levels of 10 calcium transport‐related proteins in MLE‐12 cells (A) and mouse lung tissues (B). (C–F) Representative immunofluorescence images and mean fluorescence intensity quantification of STIM1 in vitro (C, D; scale bar, 20 μm) and in vivo (E, F; scale bar, 50 μm), showing no significant differences. Data are mean ± SD. n = 3 independent samples/animals. Statistical significance was determined by one‐way ANOVA with Tukey's post hoc test (D) or unpaired two‐t [file CNS-32-e71010-s001.docx]

**Supplemental Figures and Figure Legends**

**
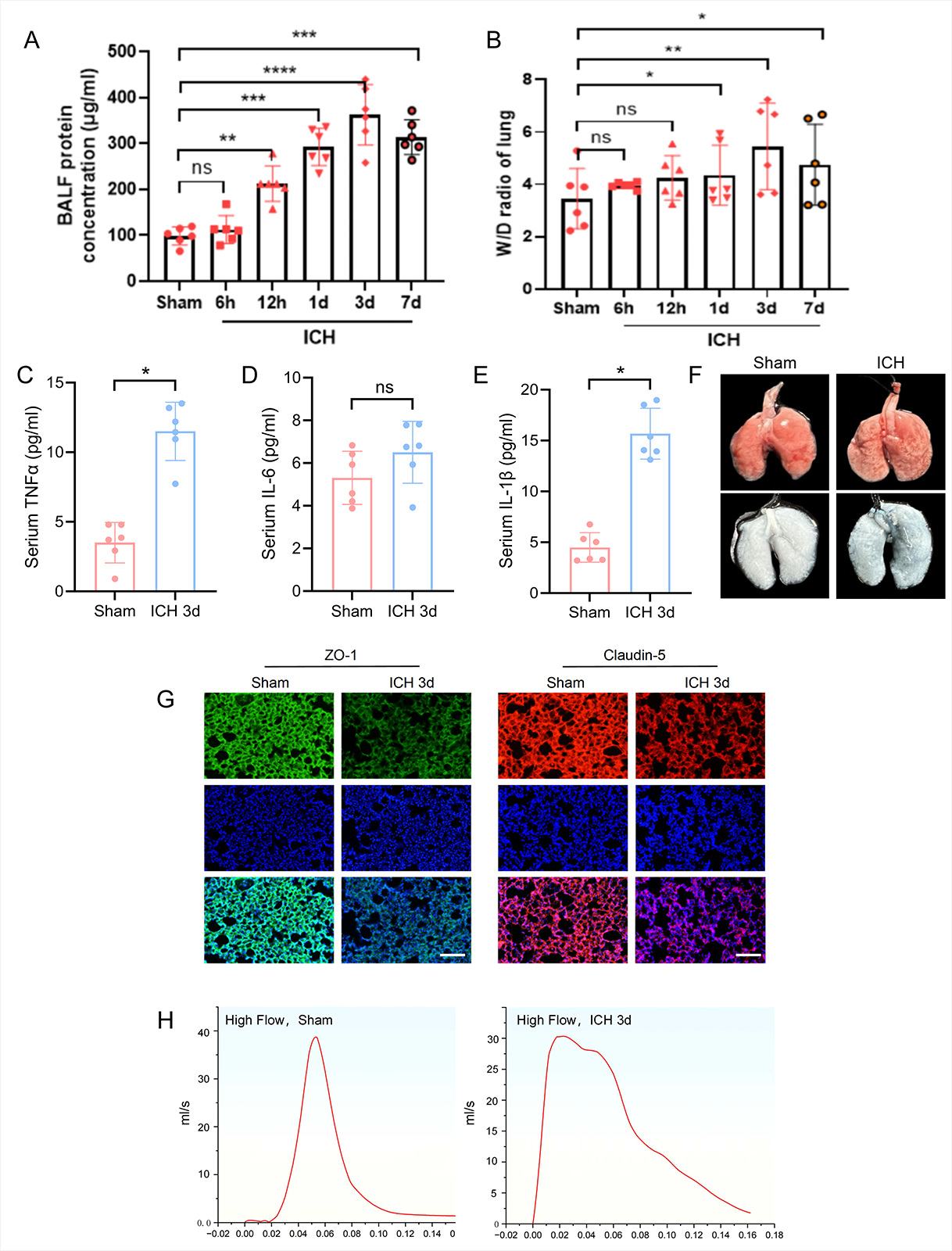
**

**Supplementary Figure 1. Extended evaluation of pulmonary edema, barrier dysfunction, and functional decline following intracerebral hemorrhage (ICH).** (A, B) Time-course evaluation of bronchoalveolar lavage fluid protein concentration (A) and lung wet/dry (W/D) ratio (B). (C-E) Serum ELISA quantification of TNF-α (C), IL-6 (D), and IL-1β (E). (F) Gross morphology of un-perfused lungs (top) and Evans blue extravasation (bottom) on day 3 post-ICH. (G) Immunofluorescence staining of tight junction proteins (ZO-1 and Claudin-5) demonstrating severe barrier disruption on day 3. Scale bar, 100 μm. (H) Assessment of pulmonary function parameters. Data are mean ± SD. n=6 independent animals. Statistical significance was determined by unpaired two-tailed Student’s *t*-test (C-E) or one-way ANOVA with Tukey’s *post hoc* test (A, B). ^*^*P* < 0.05, ^**^*P* < 0.01, ^***^*P* < 0.001, ^****^*P* < 0.0001 vs. Sham; ns, not significant.

**
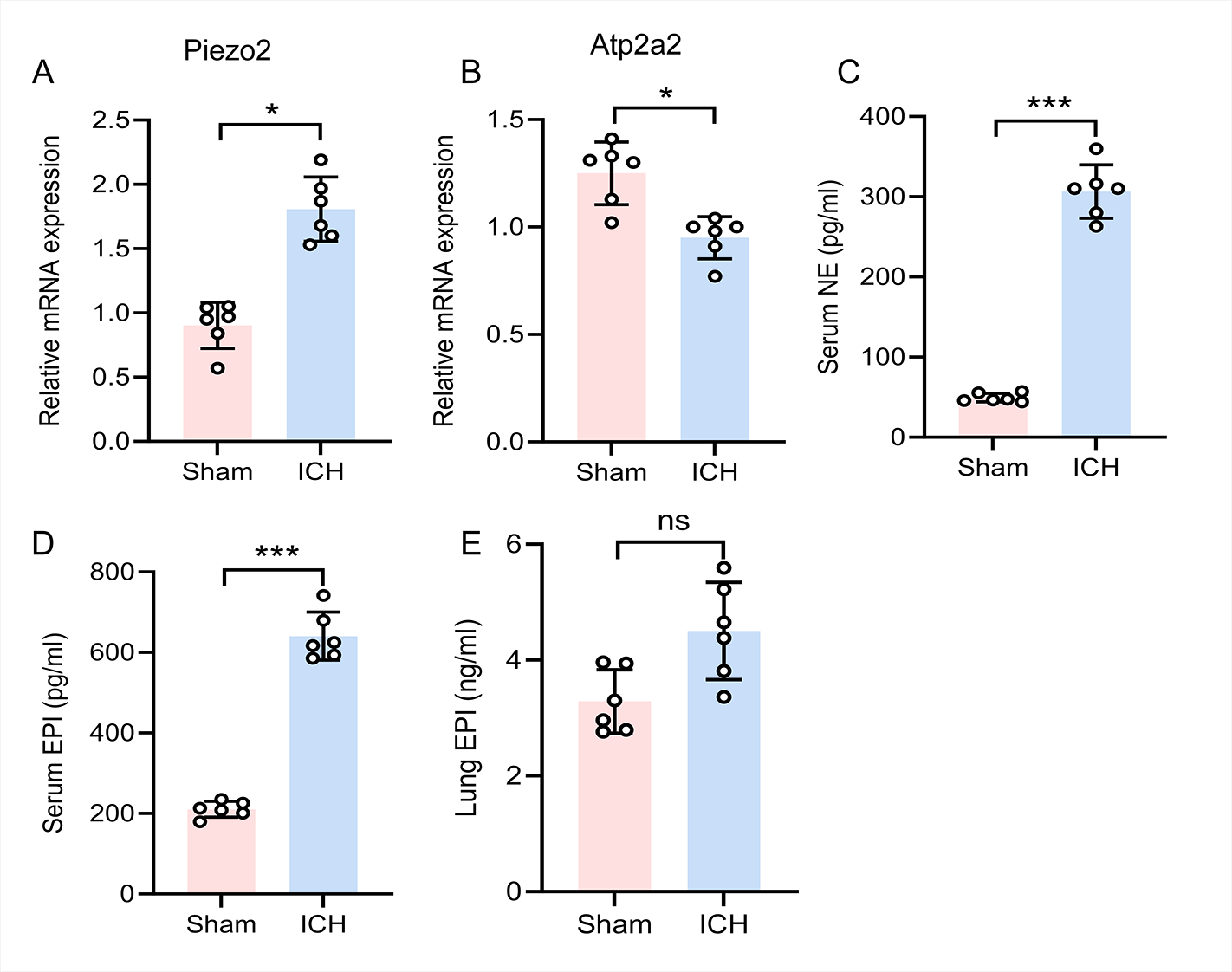
**

**Supplementary Figure 2. Systemic catecholamine surge and transcriptional alterations of calcium transporters post-intracerebral hemorrhage (ICH).** (A, B) RT-qPCR analysis demonstrating the upregulation of *Piezo2* (A) and *Atp2a2* (B) mRNA in lung tissues post-ICH. (C-E) ELISA quantification of serum Epinephrine (EPI) (C), serum NE (D), and lung EPI (E). Data are mean ± SD. n=6 independent animals. Statistical significance was determined by unpaired two-tailed Student’s *t*-test. ^*^*P* < 0.05, ^***^*P* < 0.001 vs. Sham; ns, not significant.

**
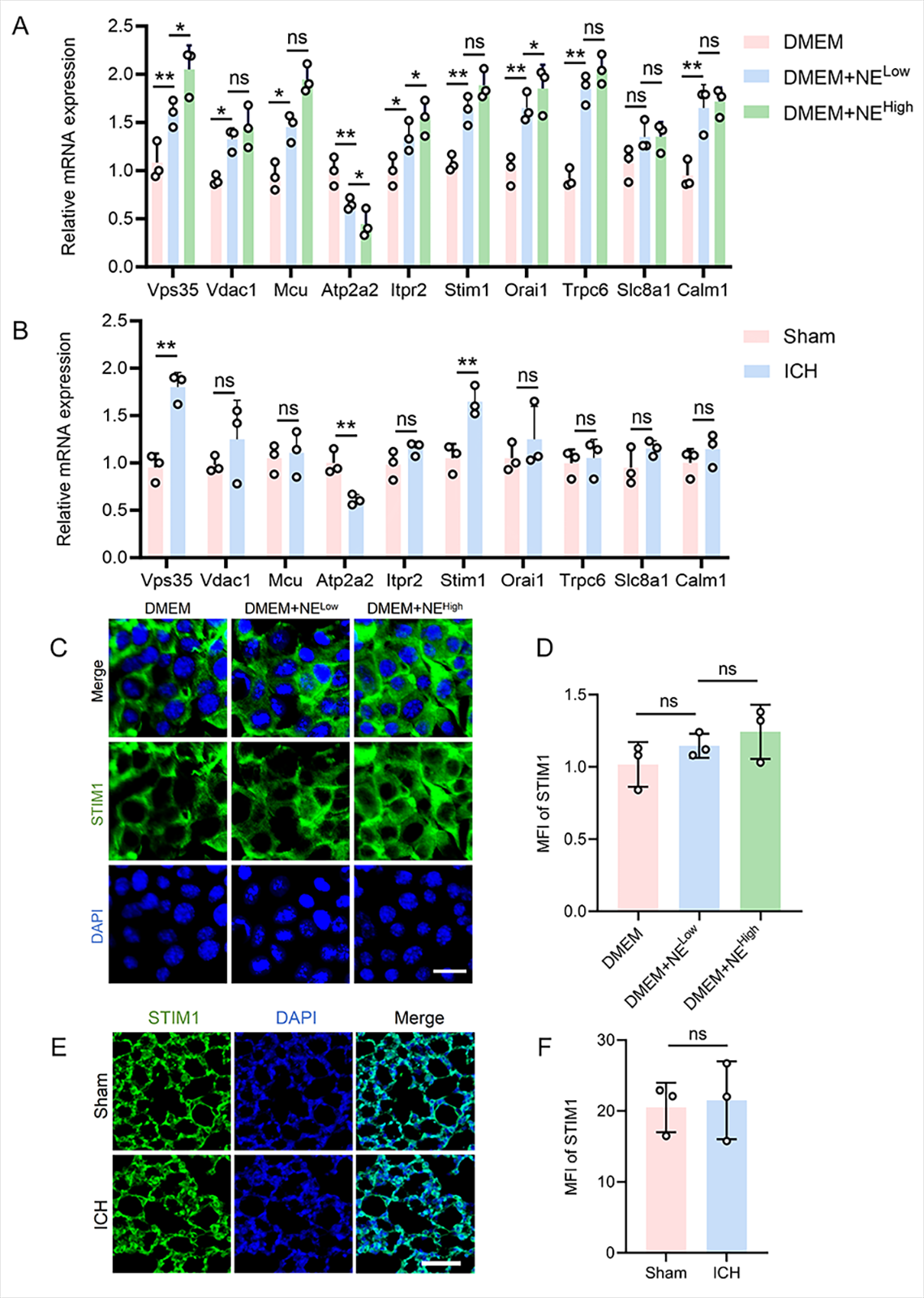
**

**Supplementary Figure 3. Sympathetic signaling inhibited calcium handling proteins.** (A, B) Validation of mRNA expression levels of 10 calcium transport-related proteins in MLE-12 cells (A) and mouse lung tissues (B). (C-F) Representative immunofluorescence images and mean fluorescence intensity quantification of STIM1 *in vitro* (C, D; scale bar, 20 μm) and *in vivo* (E, F; scale bar, 50 μm), showing no significant differences. Data are mean ± SD. n=3 independent samples/animals. Statistical significance was determined by one-way ANOVA with Tukey’s *post hoc* test (D) or unpaired two-tailed Student’s *t*-test (F). ^*^*P* < 0.05, ^**^*P* < 0.01 between indicated groups; ns, not significant.


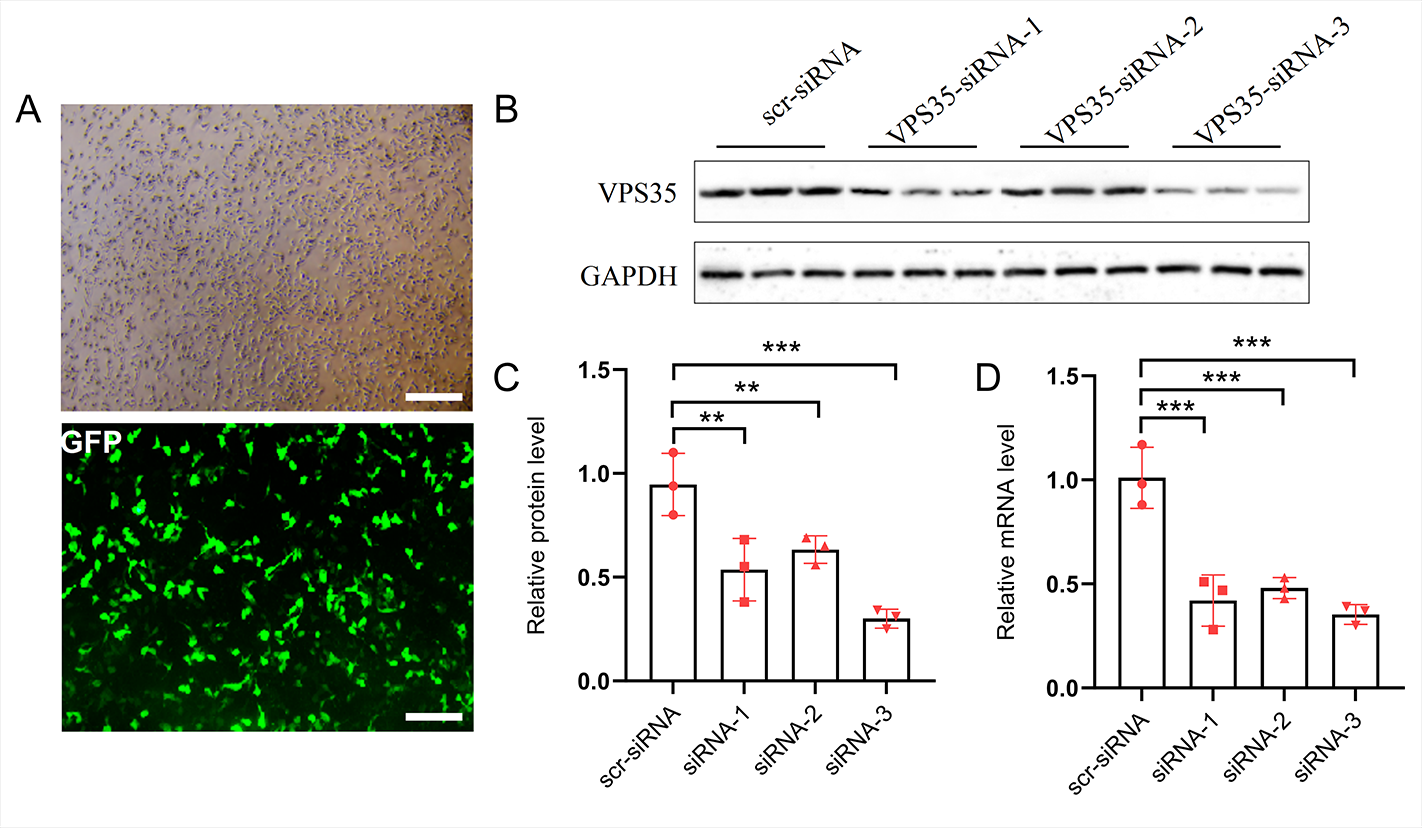


**Supplementary Figure 4. Validation of *Vps35* knockdown efficiency in MLE-12 cells.** (A) Representative bright-field (top) and GFP fluorescence (bottom) images of MLE-12 cells 24 h post-transfection. Scale bar, 200 μm. (B, C) WB images (B) and densitometric quantification (C) of VPS35 protein levels after transfection with scrambled or three distinct *Vps35*-siRNAs. (D) RT-qPCR validation of *Vps35* mRNA transcription levels. Data are mean ± SD. n=3 independent cell cultures. Statistical significance was determined by one-way ANOVA with Tukey’s *post hoc* test. ^**^*P* < 0.01, ^***^*P* < 0.001 vs. scr-siRNA.


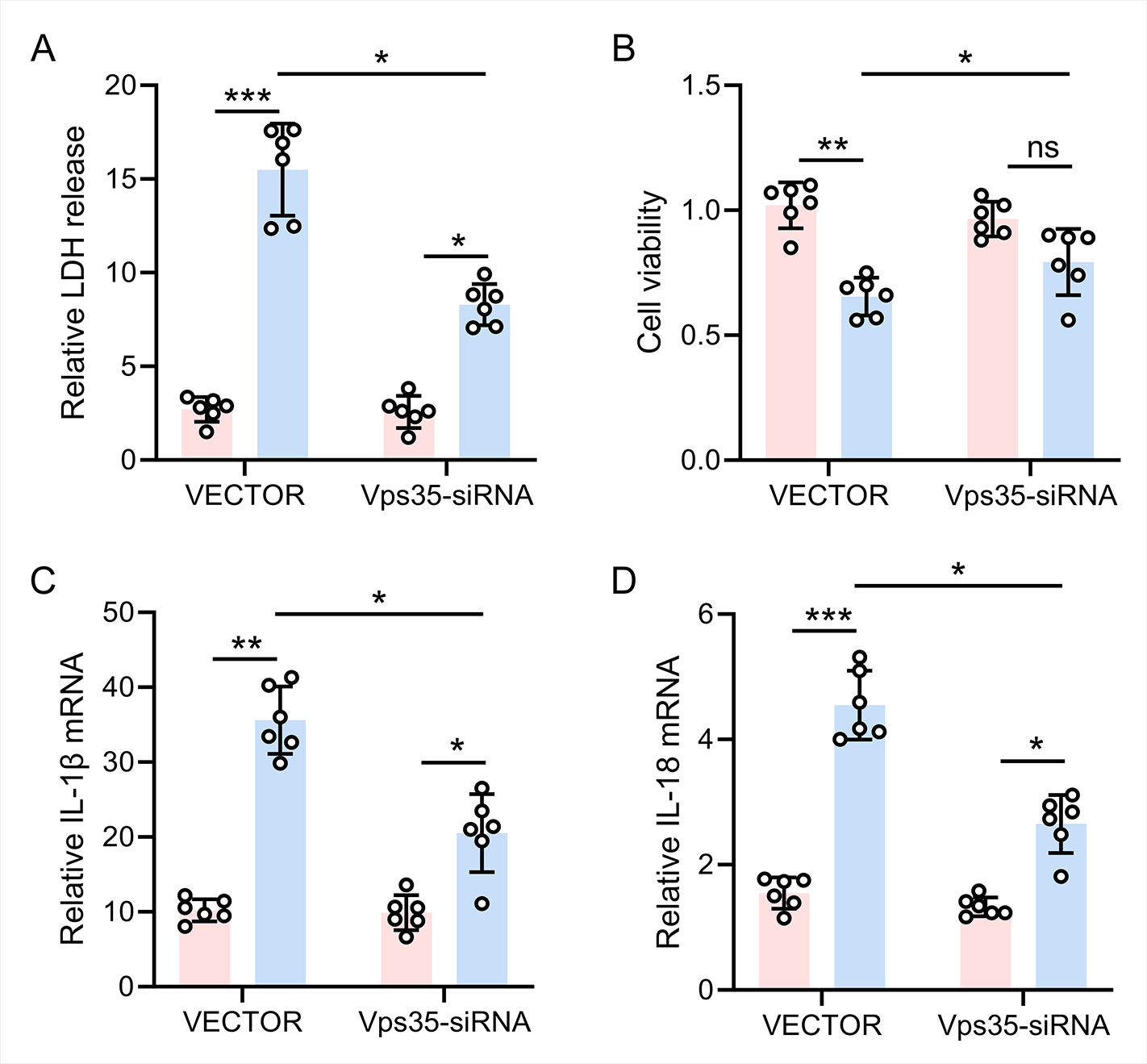


**Supplementary Figure 5. *Vps35* knockdown alleviates norepinephrine (NE)-induced cytotoxicity and pro-inflammatory cytokine transcription.** (A, B) Cytotoxicity and cell survival assessed via LDH release (A) and viability assay (B) in cells transfected with control vector or *Vps35*-siRNA, with or without NE. (C, D) RT-qPCR analysis of downstream pyroptosis-related pro-inflammatory cytokines *Il1b* (C) and *Il18* (D). Data are mean ± SD. n=6 independent cell cultures. Statistical significance was determined by one-way ANOVA with Tukey’s *post hoc* test. ^*^*P* < 0.05, ^***^*P* < 0.001 between indicated groups.


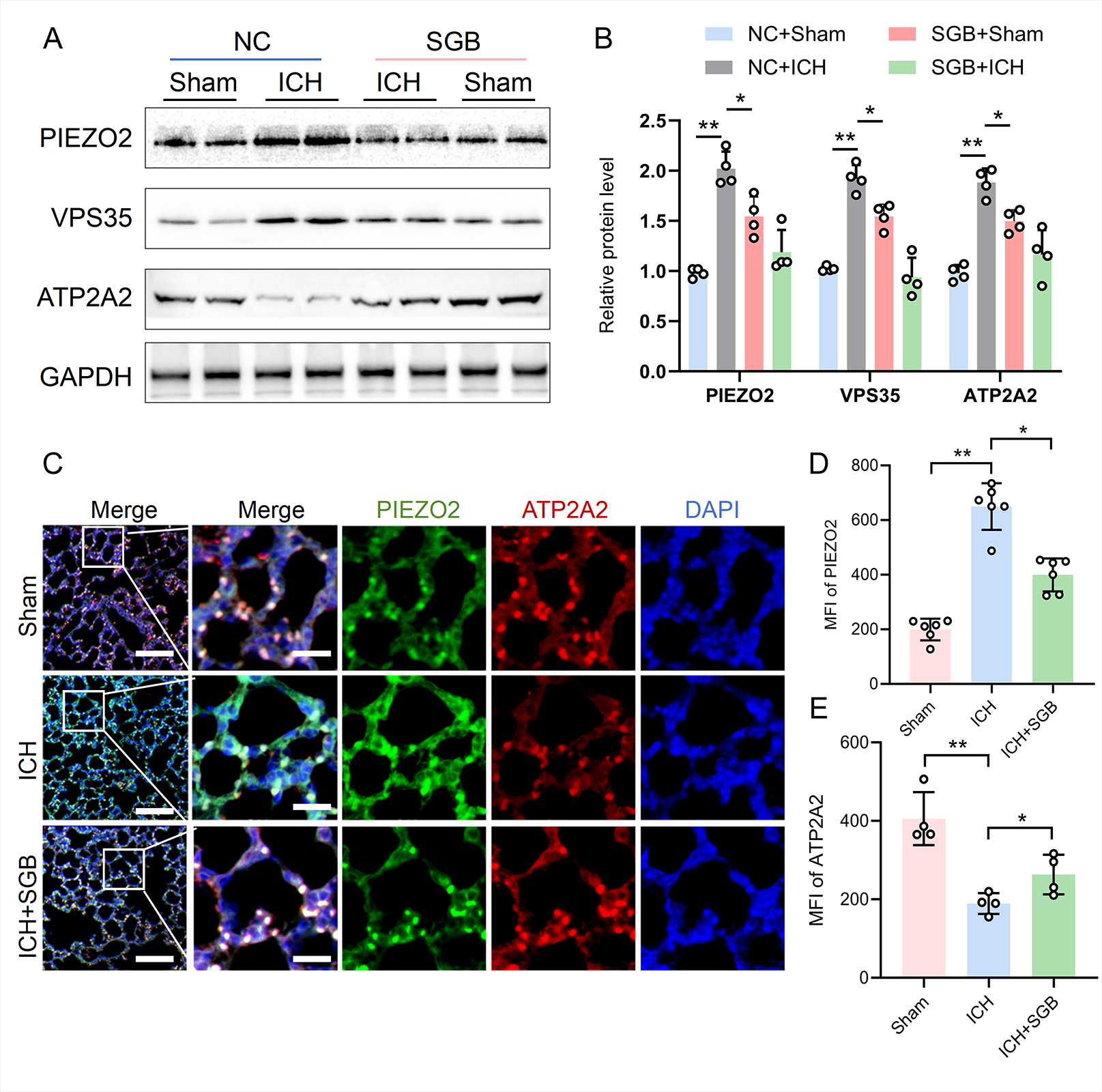


**Supplementary Figure 6. Stellate ganglion block (SGB) reverses the intracerebral hemorrhage (ICH)-induced molecular dysregulation of PIEZO2, VPS35, and ATP2A2 in the lung.** (A, B) Representative Western Blot images (A) and quantification (B) of PIEZO2, VPS35, and ATP2A2 in lung homogenates. (C-E) Representative co-immunofluorescence images of PIEZO2 (green) and ATP2A2 (red) (C) and corresponding mean fluorescence intensity quantification (D, E) among simplified experimental groups. Data are mean ± SD. n=4 (B) or n=6 (D, E) biologically independent animals. Statistical significance was determined by one-way ANOVA with Tukey’s *post hoc* test. ^*^*P* < 0.05, ^**^*P* < 0.01 between indicated groups.


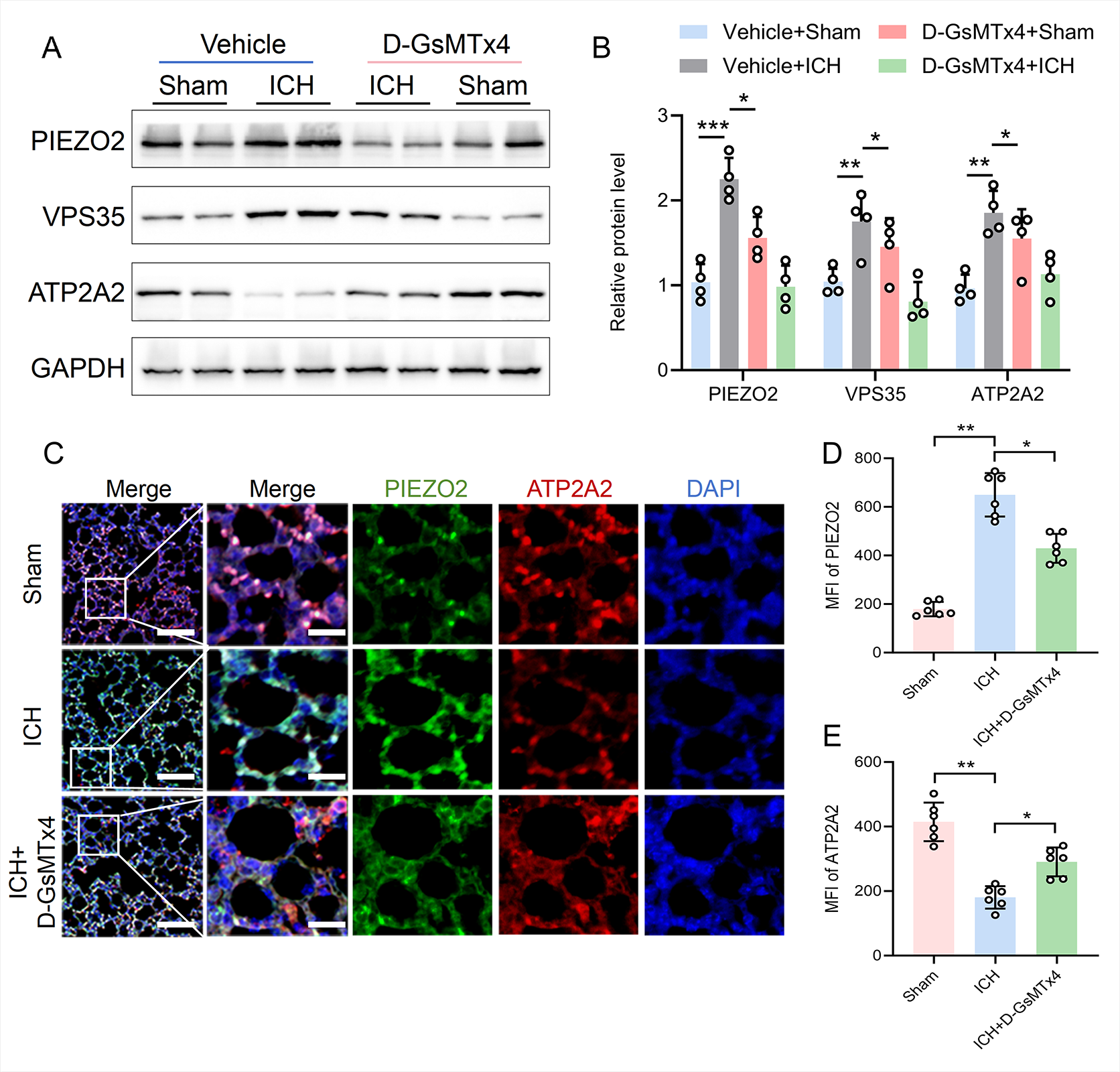


**Supplementary Figure 7. D-GsMTx4 treatment restores the expression profiles of VPS35 and ATP2A2 post-intracerebral hemorrhage (ICH).** (A, B) Immunoblotting analysis (A) and quantitative densitometry (B) of PIEZO2, VPS35, and ATP2A2 in lung tissues. (C-E) Representative co-immunofluorescence images (C) and mean fluorescence intensity quantification of PIEZO2 (D) and ATP2A2 (E), indicating that specific PIEZO2 inhibition reciprocally rescues VPS35 and ATP2A2 alterations. Data are mean ± SD. n=4 (B) or n=6 (D, E) biologically independent animals. Statistical significance was determined by one-way ANOVA with Tukey’s *post hoc* test. ^*^*P* < 0.05, ^**^*P* < 0.01, ^***^*P* < 0.001 between indicated groups.
