# Supplementary material for: Sympathetic Overactivation Drives Neurogenic Alveolar Epithelial Pyroptosis via the PIEZO2‐ER Stress Pathway in Acute Lung Injury Following Intracerebral Hemorrhage
Source: CNS Neurosci Ther. 2026 Jul 6;32(7):e71010. doi: 10.1002/cns.71010 (PMC13334374; doi:10.1002/cns.71010)
Supplement: Supplementary file 2 — Table S1: Oligonucleotide sequences of small interfering RNAs (siRNAs) for mouse Vps35 silencing. Table S2: List of primer sequences. [file CNS-32-e71010-s002.docx]

**Supplementary Methods**

**1 Study design**

This study was designed to investigate the mechanisms underlying ICH-induced ALI and to evaluate the translational potential of sympathetic blockade and targeted pharmacological interventions. To minimize potential sex-related hormonal confounding factors, such as the protective effects of estrogen, and to adhere to the 3R principles, this study utilized adult male C57BL/6J mice exclusively. Sample sizes for both in vivo and in vitro experiments were predetermined using a priori power analyses. These calculations were based on our preliminary data and comparable literature to ensure adequate statistical power (>80%) at an α level of 0.05.

The *in vivo* experiments were conducted in three sequential phases. First, in the model characterization phase, mice were assigned to Sham and ICH groups across multiple time points (6 h, 12 h, 1 d, 3 d, and 7 d). This step aimed to identify the peak window of pulmonary damage. A specific cohort (Sham versus ICH-3d) was subsequently used for transcriptomic sequencing and phenotypic assessments. Second, in the SGB intervention phase, mice were randomly allocated into four groups: Sham + NC, ICH + NC, Sham + SGB, and ICH + SGB. This phase evaluated the potential contribution of the sympathetic storm to ALI. Third, in the pharmacological intervention phase, a separate cohort was randomized into Sham + Vehicle, ICH + Vehicle, Sham + D-GsMTx4, and ICH + D-GsMTx4 groups. This arrangement was designed to examine the role of PIEZO2-mediated calcium overload. Investigators conducting the surgical modeling, behavioral tests, and histological quantification remained blinded to the group allocations.

For *in vitro* mechanistic studies, the MLE-12 was utilized in two experimental setups. In the sympathetic stimulation paradigm, cells were divided into a vehicle control group (DMEM), a low-dose norepinephrine group (DMEM + NE^Low^, 1 μM), and a high-dose norepinephrine group (DMEM + NE^High^, 10 μM). In the genetic knockdown paradigm, cells were assigned to four groups to investigate the role of Vps35. These groups included Vector + DMEM, Vector + DMEM + NE (10 μM), Vps35-siRNA + DMEM, and Vps35-siRNA + DMEM + NE (10 μM).

**2 Experimental animals**

All animal experiments and protocols were approved by the Animal Ethics Committee of Yangzhou University School of Medicine (Approval No. 20250315). The study was conducted in accordance with the National Institutes of Health Guide for the Care and Use of Laboratory Animals. Adult male C57BL/6J mice, weighing 20-25 g, were obtained from the Animal Experimental Center of Yangzhou University. Animals were housed under a standard 12-hour light/dark cycle and provided ad libitum access to food and water. Mice were assigned to experimental groups using a random number generator. The investigators performing outcome assessments were blinded to these group allocations.

**3 Intracerebral hemorrhage model**

To induce the murine ICH model, mice were subjected to a stereotaxic infusion of bacterial collagenase type IV [1]. Mice were first anesthetized via 2% isoflurane inhalation and subsequently secured in a stereotaxic apparatus (Model 68801, RWD Life Science, Shenzhen, China). A micro-burr hole was drilled over the right hemisphere after exposing the skull. A Hamilton syringe was then advanced into the right basal ganglia. The stereotaxic coordinates were 0.2 mm posterior and 2.2 mm lateral to the bregma, at a depth of 3.5 mm from the dura. A total volume of 0.3 μL saline containing 0.03 U of collagenase was microinjected at a constant rate of 0.06 μL/min. To minimize potential solution reflux along the needle tract, the syringe was left in situ for 10 minutes before gradual withdrawal. Mice in the sham group underwent identical surgical procedures but received an equal volume of phosphate-buffered saline (PBS). To assess the temporal profile of pulmonary damage, lung tissues were collected and evaluated at various intervals (6 h, 12 h, 1 d, 3 d, and 7 d) post-hemorrhage.

**4 Stellate ganglion block (SGB)**

To investigate the role of the sympathetic nervous system in ICH-induced lung injury, mice were randomly allocated into four experimental groups: Sham+NC, ICH+NC, Sham+SGB, and ICH+SGB. The SGB procedure was performed at 1 hour, 24 hours, and 48 hours post-ICH induction. Mice were anesthetized with 2% isoflurane and positioned prone on a surgical platform. The cartilaginous tip of the seventh cervical vertebra (C7) spinous process was identified by palpation. A microsyringe needle was advanced anteriorly along the right parasagittal plane of C7. When a loss of resistance was felt, suggesting the needle tip had bypassed the vertebral body, the needle was slightly withdrawn by approximately 0.5 mm. After aspiration to confirm the absence of blood or cerebrospinal fluid, 40 μL of 0.25% ropivacaine was slowly injected into the target region to induce sympathetic blockade. Mice in the NC groups received an equivalent volume of normal saline. Successful SGB was confirmed upon recovery from anesthesia by the presence of unilateral Horner's syndrome on the ipsilateral side. This syndrome, characterized by ptosis, enophthalmos, and miosis [2], was evaluated as a qualitative “yes/no” assessment by visually comparing the ipsilateral eye with the contralateral unaffected eye. Mice failing to exhibit these obvious clinical signs were considered to have unsuccessful blocks and were excluded from subsequent analyses.

**5 PIEZO2 inhibition (D-GsMTx4)**

To inhibit PIEZO2 in vivo, D-GsMTx4, a widely used mechanosensitive ion channel inhibitor, was administered intraperitoneally (i.p.) at a dose of 0.5 mg/kg (or vehicle) at 1, 24, and 48 h post-ICH. This specific dosage and administration route were selected based on recent in vivo studies demonstrating its efficacy and safety in systemically modulating PIEZO-dependent signaling [3], as well as its established application in targeting PIEZO2-related neural injury models [1]. Detailed information regarding the drug formulation and administration is provided in the Supplementary Methods.

**6 Morris Water Maze (MWM) test**

Spatial learning and memory were assessed using the MWM test at 14 days post-ICH. The apparatus consisted of a circular pool with a diameter of 120 cm. The pool was filled with opaque water maintained at 22 ± 1°C and conceptually divided into four equal quadrants. A hidden platform, 10 cm in diameter, was submerged 0.8 cm below the water surface in the target quadrant. An automated video tracking system (VisuTrack 2.0, Xinruan Co., Shanghai, China) recorded the movements of the mice. The assessed parameters included escape latency, time spent in the target quadrant, the number of platform crossings, and average swimming speed. Escape latency was defined as the time taken to find the hidden platform [4].

**7 Electrocardiography and heart rate variability**

To continuously monitor cardiac electrical activity and assess autonomic nervous system alterations, a non-invasive rodent electrocardiogram (ECG) system (Model 3306B, Shanghai Yuyan Instruments Co., Ltd., Shanghai, China) was utilized. Following ICH induction, electrocardiographic data were acquired and processed to determine the heart rate (HR). Specifically, the power spectrum was integrated into low-frequency (LF, 0.20–0.75 Hz) and high-frequency (HF, 0.75–2.5 Hz) bands [5]. The HF power was calculated to quantitatively assess the balance between parasympathetic and sympathetic autonomic tones. This analysis helped evaluate the potential presence of sympathetic hyperactivity.

**8 Pulmonary function testing**

On day 3 post-ICH, pulmonary mechanics and airway function were systematically evaluated using a small animal pulmonary function testing system (DSI Buxco® PFT, Beijing Bai An Ji Technology Co., Ltd., Beijing, China). Following deep anesthesia and orotracheal intubation, the mice were connected to the apparatus. Respiratory parameters, including airway resistance and dynamic compliance, were continuously recorded. These data were subsequently analyzed to evaluate the severity of ICH-induced pulmonary impairment.

**9 Chest X-ray imaging**

To radiographically evaluate lung pathology, chest X-rays were acquired using an IVIS Lumina XRMS in vivo imaging system (Revvity, Waltham, MA, USA). Mice were anesthetized via inhalation of 2% isoflurane to minimize movement artifacts. They were then positioned supine with their limbs gently extended to provide adequate exposure of the thoracic cavity. Radiographic images were captured using an X-ray tube voltage of 45 kV, a current of 250 μA, and an exposure time of 2 s. The obtained radiographs were subsequently analyzed to assess structural lung abnormalities.

**10 Brain water content and lung W/D ratio**

Mice were euthanized, and the brains were immediately extracted. The right hemisphere was weighed to obtain the wet weight, and subsequently dehydrated in an oven at 80°C for 24 h to determine the dry weight. Brain water content was calculated using the formula: (wet weight - dry weight) / wet weight × 100%. A similar protocol was applied to calculate the wet/dry (W/D) weight ratio of lung tissues for pulmonary edema evaluation.

**11 Lung Permeability**

To evaluate pulmonary vascular permeability, mice received a tail-vein injection of 2% Evans blue (EB) dye (4 mL/kg). Following a 2-hour circulation period, animals underwent transcardial saline perfusion to remove intravascular dye. Lungs were then excised, photographed, and processed for EB extraction and quantification. To further assess alveolar-capillary barrier function, the total protein concentration in bronchoalveolar lavage fluid (BALF) was measured using a BCA assay kit (Cat#PC0020, Solarbio, Beijing, China).

**12 Histology**

Brain and lung tissues were fixed in 4% paraformaldehyde, embedded in paraffin, and cut into 5-μm sections. Brain sections were stained with Hematoxylin and Eosin (HE) and Nissl, while lung sections were stained with HE alone. Lung injury was evaluated in a blinded manner using a standardized scoring system. This system incorporated assessments of alveolar congestion, hemorrhage, neutrophil infiltration, and alveolar wall thickening.

**13 RNA sequencing and bioinformatics analysis**

To investigate transcriptomic alterations in the lungs following ICH, total RNA was extracted from pulmonary tissues of the Sham and 3-day post-ICH groups using TRIzol reagent (Cat# R1100, Solarbio, Beijing, China). RNA integrity and concentration were assessed using an Agilent 2100 Bioanalyzer. Library construction and paired-end sequencing were performed on an Illumina NovaSeq platform (Majorbio Bio-Pharm Technology Co., Ltd., Shanghai, China). Differential expression analysis was conducted using the DESeq2 R package. Differentially expressed genes (DEGs) were identified based on an adjusted P-value < 0.05 and a |log2(fold change)| > 1. The expression profiles of these DEGs were visualized via volcano plots and hierarchical clustering heatmaps. Finally, Gene Ontology (GO) and Kyoto Encyclopedia of Genes and Genomes (KEGG) enrichment analyses were conducted to characterize the biological processes and signaling pathways associated with ICH.

**14 Cell culture and *in vitro* sympathetic stimulation**

The mouse alveolar epithelial cell line MLE-12 (Cat# SNL-414, Sunncell, Wuhan, China) was cultured in Dulbecco’s Modified Eagle Medium (DMEM; Cat# 11965092, Gibco, Grand Island, NY, USA) supplemented with 10% fetal bovine serum (FBS; Cat# A5256701, Gibco). Cells were maintained in a 37°C humidified incubator with 5% CO₂. To simulate sympathetic activation *in vitro*, MLE-12 cells were treated with norepinephrine (NE; Cat# HY-N7142S3, MedChemExpress, NJ, USA). Cells were divided into three treatment conditions: complete medium alone (DMEM), 1 μM NE (DMEM + NE^Low^), or 10 μM NE (DMEM + NE^High^).

**15 siRNA transfection**

For Vps35 silencing, cells were transfected with one of three Vps35-specific small interfering RNAs (si-Vps35 1-3) or a GFP-conjugated scrambled negative control siRNA (Scr-siRNA), all synthesized by GeneChem (Shanghai, China). Transfections were performed using Lipofectamine 3000 (Cat# L3000008, Solarbio, Beijing, China) according to the manufacturer's instructions. The specific oligonucleotide sequences are listed in Supplementary Table 1. Transfection efficiency was assessed 24 hours post-transfection using GFP fluorescence microscopy. Knockdown efficiency was subsequently validated via quantitative RT-PCR and Western blotting.

Supplementary Table 1. Oligonucleotide sequences of small interfering RNAs (siRNAs) for mouse *Vps35* silencing.

| siRNA Name | Target Gene | Sequence (5’ to 3’) |
| --- | --- | --- |
| si-Vps35-1 | Mouse *Vps35* | Sense: GCUACUGUUGCUUACAAUUTT Antisense: AAUUGUAAGCAACAGUAGCTT |
| si-Vps35-2 | Mouse *Vps35* | Sense: GCAUGGAGCUUGAGUAUAATT Antisense: UUAUACUCAAGCUCCAUGCTT |
| si-Vps35-3 | Mouse *Vps35* | Sense: GGATCGAAGUGGUUCUGAATT Antisense: UUCAGAACCACUUCGAUCCTT |
| Scr-siRNA | Negative Control | Sense: UUCUCCGAACGUGUCACGUTT Antisense: ACGUGACACGUUCGGAGAATT |

**16 Flow cytometric analysis of intracellular calcium**

To evaluate intracellular calcium (Ca²⁺) levels, MLE-12 cells were labeled with the Ca²⁺-sensitive fluorescent probe Fluo-3 AM (Cat# S1056, Beyotime Biotechnology, Shanghai, China). Post-treatment cells were harvested and incubated with 3 μM Fluo-3 AM for 45 min at 37°C in the dark. Cells were then washed with PBS to remove excess extracellular probe. Intracellular fluorescence intensity was acquired and analyzed using a CytoFLEX flow cytometer (Beckman Coulter, Brea, CA, USA).

**17 Colorimetric calcium assay**

Total calcium levels in lung tissue homogenates and MLE-12 cell lysates were quantitatively measured using a Calcium Colorimetric Assay Kit (Cat# S1063S, Beyotime Biotechnology, Shanghai, China). All assays were performed according to the manufacturer’s instructions.

**18 Quantification of cytokines and catecholamines**

Blood serum, whole brain, and lung tissues were collected and processed. Concentrations of pro-inflammatory cytokines, including TNF-α (Cat# ml002095), IL-6 (Cat# ml063159), and IL-1β (Cat# ml106733), were quantified using ELISA kits from mlbio (Shanghai, China). The levels of sympathetic neurotransmitters, specifically norepinephrine (Cat# CB10569-Mu) and epinephrine (EPI) (Cat# CB10609-Mu), were measured using corresponding ELISA kits from Coibo Bio (Shanghai, China). Absorbance readings and sample preparations were conducted according to the manufacturers' protocols.

**19 Cell viability and pyroptosis assessment**

To evaluate cytotoxicity in MLE-12 cells, the release of lactate dehydrogenase (LDH) into the culture supernatant was measured using an LDH Cytotoxicity Assay Kit (Cat# C0019S, Beyotime Biotechnology, Shanghai, China). Cell viability was assessed using the Cell Counting Kit-8 (CCK-8; Cat# C0037, Beyotime Biotechnology). Both assays were performed following the manufacturer's instructions, and optical densities were recorded using a microplate reader.

**20 Immunofluorescence staining**

Fixed tissue sections and MLE-12 cells were permeabilized with 0.1% Triton X-100 and blocked with 5% BSA for 1 h at room temperature. Samples were then incubated overnight at 4°C with the following primary antibodies: anti-Piezo2 (1:50; Cat# AB_2718829, Thermo Fisher) and a panel of Proteintech antibodies (all diluted at 1:250), including anti-ATP2A2 (Cat# 27311-1-AP), anti-C/EBP homologous protein (CHOP) (Cat# 15204-1-AP), anti-NLRP3 (Cat# 30109-1-AP), anti-ASC (Cat# 10500-1-AP), anti-tyrosine hydroxylase (TH) (Cat# 25859-1-AP), anti-VPS35 (Cat# 10236-1-AP), anti-STIM1 (Cat# 11565-1-AP), anti-ZO-1 (Cat# 21773-1-AP), and anti-Claudin-5 (Cat# 29767-1-AP). After washing, samples were incubated with Alexa Fluor-conjugated secondary antibodies (1:1000; Cat# 4412S or 4410S, Cell Signaling Technology) for 1 h in the dark. Nuclei were counterstained with DAPI. Images were captured using a Zeiss confocal microscope and quantified with ImageJ software.

**21 RT-qPCR analysis**

Total RNA from lung tissues and MLE-12 cells was extracted using TRIzol reagent. RNA was subsequently reverse-transcribed into cDNA using the FastKing RT Kit (Cat# KR118-01, TIANGEN Biotech, Beijing, China). RT-qPCR was performed using SYBR Green Master Mix on a QuantStudio Real-Time PCR System (Thermo Fisher Scientific, Waltham, MA, USA). The relative mRNA expression levels of target genes (*Il1b, Il18, Piezo2, Atp2a2, Vps35*, and 10 calcium transport-related genes) were calculated using the 2^−ΔΔCt^ method. Gapdh served as the internal normalization control. All specific primer sequences are detailed in Supplementary Table S2.

Supplementary Table 2. List of primer sequences.

| Gene | Primer sequence(Forward) | Primer sequence(Reverse） |
| --- | --- | --- |
| *Il1b* | GCAACTGTTCCTGAACTCAACT | ATCTTTTGGGGTCCGTCAACT |
| *Il18* | GACTCTTGCGTCAACTTCAAGG | CAGGCTGTCTTTTGTCAACGA |
| *Piezo2* | GCTGGAGATGTTCATCGAAGTG | GCACTGTTCAGCGTCATATCTC |
| *Atp2a2* | CTCCTTGCCCGTGATTCTCA | CCAGCAGGTCCTGGTCATC |
| *Vps35* | GCTACGTTCTGATGATCCTGACCAG | AAGGTGGCAGTGTGAAGCGAATC |
| *Vdac1* | ACAAAGCTGCCGTCGACTTT | GTTTGTCGCCGCATCAATCT |
| *Mcu* | AAAGGAGCCAAAAAGTCGTGC | TCATGAGGCAGGTGTCTAGGT |
| *Itpr2* | CTTGGTCAGCGTGCTGGTA | TCATAGCCACTGGGAGACAG |
| *Orai1* | CGTAACTCTATGCGCTCATGAT | CCGAGGCTTCTAGGTAGCATT |
| *Trpc6* | AAGTGAACCCCCACGTAGAT | TGCCTGACCATGAGAGCAAA |
| *Slc8a1* | TGGAACAAGACTCCGCAGTA | AACGCCAGCGTAGAGTTTTG |
| *Calm1* | GACCATGATGGTCGCGTTAA | CTCGAGAGTGATTCCTCCCAT |
| *Stim1* | TTTTGTGGTGGTCGTGAATGA | CCTGGATACAGCCTGCCTTC |
| *Gapdh* | TGTGTCCGTCGTGGATCTGA | CCTGCTTCACCACCTTCTTGA |

**22Western blotting**

Total proteins were extracted using RIPA lysis buffer containing protease inhibitors. Protein concentrations were determined using a BCA assay kit. Equal protein amounts (20 μg) were separated via SDS-PAGE and transferred onto PVDF membranes. Membranes were blocked with 5% non-fat milk for 1 h at room temperature. Primary antibody incubation was performed overnight at 4°C using: anti-PIEZO2 (1:1000; Cat# AB_2718829, Thermo Fisher); anti-cleaved Caspase-1 (1:2000; Cat# YM9369) and anti-cleaved GSDMD (N-terminal, 1:2000; Cat# YM8489) from ImmunoWay Biotechnology. Additional Proteintech antibodies included anti-NLRP3 (1:2000; Cat# 30109-1-AP), anti-total Caspase-1 (1:2000, Cat# 22915-1-AP), anti-full-length GSDMD (1:5000; Cat# 20770-1-AP), anti-VPS35 (1:5000; Cat# 10236-1-AP), anti-ATP2A2 (1:5000; Cat# 27311-1-AP), anti-Tubulin (1:2000; Cat# 10094-1-AP), and anti-GAPDH (1:50,000; Cat# 60004-1-Ig). Membranes were subsequently incubated with HRP-conjugated secondary antibodies (1:3000; Cat# RGAR001 or RGAM001, Proteintech) for 1 h at room temperature. Protein bands were visualized using an enhanced chemiluminescence detection system and quantified with ImageJ software.

**23 Statistical analysis**

Statistical analyses were performed using GraphPad Prism 10.0 (GraphPad Software, USA). Data are presented as mean ± standard deviation (SD). Prior to parametric testing, data normality and variance homogeneity were evaluated using the Shapiro-Wilk test and Levene’s (or Brown-Forsythe) test, respectively. Comparisons between two independent groups were analyzed using an unpaired two-tailed Student’s t-test. For multiple group comparisons with a single independent variable, a one-way analysis of variance (ANOVA) followed by Tukey’s *post hoc* test was applied. Time-course behavioral data were analyzed using a two-way repeated-measures ANOVA followed by Bonferroni’s *post hoc* test. Sample size (n) represents the number of independent biological replicates per group. A *P*-value < 0.05 was considered statistically significant.

**Reference**

1. Xiao Y, Zhang Y, Yuan W, et al. Piezo2 Contributes to Traumatic Brain Injury by Activating the RhoA/ROCK1 Pathways. Mol Neurobiol. 2024;61(10):7419-7430. doi:10.1007/s12035-024-04058-y
2. Shi ZM, Jing JJ, Xue ZJ, et al. Stellate ganglion block ameliorated central post-stroke pain with comorbid anxiety and depression through inhibiting HIF-1α/NLRP3 signaling following thalamic hemorrhagic stroke. J Neuroinflammation. 2023;20(1):82. Published 2023 Mar 21. doi:10.1186/s12974-023-02765-2
3. He H, Zhou J, Cao S, Liu W, Mei Z, Liu M. Electroacupuncture attenuates intestinal epithelial ferroptosis in inflammatory bowel disease via Piezo1-mediated mitochondrial homeostasis. Chin Med. 2025;20(1):161. Published 2025 Oct 6. doi:10.1186/s13020-025-01218-7
4. Jiang T, Wang J, Wang Y, et al. Mitochondrial protein prohibitin promotes learning memory recovery in mice following intracerebral hemorrhage via CAMKII/CRMP signaling pathway. Neurochem Int. 2023;171:105637. doi:10.1016/j.neuint.2023.105637
5. Cai W, Xu D, Zeng C, et al. Modulating Lysine Crotonylation in Cardiomyocytes Improves Myocardial Outcomes. Circ Res. 2022;131(5):456-472. doi:10.1161/CIRCRESAHA.122.321054
